# Phospholemman Phosphorylation Regulates Vascular Tone, Blood Pressure, and Hypertension in Mice and Humans

**DOI:** 10.1161/CIRCULATIONAHA.119.040557

**Published:** 2020-12-18

**Authors:** Andrii Boguslavskyi, Sergiy Tokar, Oleksandra Prysyazhna, Olena Rudyk, David Sanchez-Tatay, Hamish A.L. Lemmey, Kim A. Dora, Christopher J. Garland, Helen R. Warren, Alexander Doney, Colin N.A. Palmer, Mark J. Caulfield, Julia Vlachaki Walker, Jacqueline Howie, William Fuller, Michael J. Shattock

**Affiliations:** British Heart Foundation Centre of Research Excellence, King’s College London, United Kingdom (A.B., S.T., O.P., O.R., D.S.-T., M.J.S.). Clinical Pharmacology, The William Harvey Research Institute (O.P., H.R.W., M.J.C.), National Institute for Health Research, Biomedical Research Centre (H.R.W., M.J.C.), Barts and The London School of Medicine and Dentistry, Queen Mary University of London, United Kingdom. Department of Pharmacology, University of Oxford, United Kingdom (H.A.L.L., K.A.D., C.J.G.). Medicines Monitoring Unit, School of Medicine (A.D.), Division of Cardiovascular and Diabetes Medicine (C.N.A.), University of Dundee, United Kingdom. Institute of Cardiovascular and Medical Sciences, University of Glasgow, United Kingdom (J.V.W., J.H., W.F.).

**Keywords:** blood, pressure, hypertension, Na/K ATPase, phospholemman, vascular smooth muscle, vascular tone

## Abstract

Although it has long been recognized that smooth muscle Na/K ATPase modulates vascular tone and blood pressure (BP), the role of its accessory protein phospholemman has not been characterized. The aim of this study was to test the hypothesis that phospholemman phosphorylation regulates vascular tone in vitro and that this mechanism plays an important role in modulation of vascular function and BP in experimental models in vivo and in humans.

**Methods:**

In mouse studies, phospholemman knock-in mice (PLM^3SA^; phospholemman [FXYD1] in which the 3 phosphorylation sites on serines 63, 68, and 69 are mutated to alanines), in which phospholemman is rendered unphosphorylatable, were used to assess the role of phospholemman phosphorylation in vitro in aortic and mesenteric vessels using wire myography and membrane potential measurements. In vivo BP and regional blood flow were assessed using Doppler flow and telemetry in young (14–16 weeks) and old (57–60 weeks) wild-type and transgenic mice. In human studies, we searched human genomic databases for mutations in phospholemman in the region of the phosphorylation sites and performed analyses within 2 human data cohorts (UK Biobank and GoDARTS [Genetics of Diabetes Audit and Research in Tayside]) to assess the impact of an identified single nucleotide polymorphism on BP. This single nucleotide polymorphism was expressed in human embryonic kidney cells, and its effect on phospholemman phosphorylation was determined using Western blotting.

**Results:**

Phospholemman phosphorylation at Ser63 and Ser68 limited vascular constriction in response to phenylephrine. This effect was blocked by ouabain. Prevention of phospholemman phosphorylation in the PLM^3SA^ mouse profoundly enhanced vascular responses to phenylephrine both in vitro and in vivo. In aging wild-type mice, phospholemman was hypophosphorylated, and this correlated with the development of aging-induced essential hypertension. In humans, we identified a nonsynonymous coding variant, single nucleotide polymorphism rs61753924, which causes the substitution R70C in phospholemman. In human embryonic kidney cells, the R70C mutation prevented phospholemman phosphorylation at Ser68. This variant’s rare allele is significantly associated with increased BP in middle-aged men.

**Conclusions:**

These studies demonstrate the importance of phospholemman phosphorylation in the regulation of vascular tone and BP and suggest a novel mechanism, and therapeutic target, for aging-induced essential hypertension in humans.

## Abstract 2

Supplemental Digital Content is available in the text.

**Clinical Perspective**

**What Is New?**

- Despite many previous studies implicating smooth muscle Na/K ATPase and endogenous cardiotonic steroids in hypertension, this is the first study demonstrating a role for its principle regulatory accessory protein phospholemman (PLM).
- Phosphorylation of PLM profoundly influences vascular tone and blood pressure regulation in mice, and its hypophosphorylation is associated with aging-induced essential hypertension.
- In humans, a single nucleotide polymorphism was identified in PLM exon 10 that results in an R70C mutation that in vitro leads to PLM hypophosphorylation and, in 2 human cohorts (UK Biobank and GoDARTS [Genetics of Diabetes Audit and Research in Tayside]), is associated with a significant elevation of blood pressure in middle-aged men.

**What Are the Clinical Implications?**

- Interventions that prevent PLM dephosphorylation and Na/K ATPase downregulation may provide new therapeutic approaches to treatment of essential hypertension.
- Existing interventions that prevent downregulation of signaling pathways that terminate in PLM phosphorylation (for example, β-blockers) may in part exert their beneficial antihypertensive effect through this mechanism.
- The age- and sex-dependence of these observations needs further investigation.

The Na/K ATPase is ubiquitously expressed and maintains the Na and K transmembrane gradients essential for a plethora of cell functions including electric excitability, secondary active transport, and cell signaling.^1–3^

In vascular smooth muscle cells, the Na/K ATPase (specifically the α2 isoform) plays an important role in modulating vascular smooth muscle cell tone and blood pressure (BP).^1,2,4–7^ The hypertensive effects of Na/K pump inhibition with both exogenous and endogenous cardiotonic steroids have long been recognized.^8–10^ Agents that stimulate the pump are hypotensive, whereas agents that inhibit the pump (and specifically the α2 subunit in vascular smooth muscle) are hypertensive.^8,9,11^ Endogenous cardiotonic steroids, for example, play a role in setting BP,^12^ and exogenous ouabain completely blocks classical acetylcholine-induced nitric oxide (NO)–mediated vasodilation^13^ and substantially blocks reactive hyperemia^14^—suggesting that Na/K ATPase activity not only influences vascular contractility but also is central to the action of endogenous vasodilators.^4,15–19^

Despite overwhelming evidence for a role of the Na/K ATPase in regulation of smooth muscle tone and BP, surprisingly little is known about the role of phospholemman, the muscle-specific regulator of Na/K ATPase, in modulating BP, and even less is known about its possible contribution to hypertension. Phospholemman (FXYD1) is a member of the FXYD family of Na/K ATPase modulatory proteins and in cardiac muscle links cellular signaling pathways (specifically protein kinase C [PKC] and protein kinase A [PKA]) to Na/K pump activation.^20^ In the heart, phosphorylation of phospholemman disinhibits the Na/K ATPase by lowering its affinity for Na (K_m_) and by increasing its maximal activity (V_max_) and hence increases Na efflux in response to hormonal stimulation and heart rate elevation.^21–23^

Although phospholemman is expressed in vascular smooth muscle, its role in regulating Na/K ATPase in this tissue has not been established, and the limited literature is incomplete and contradictory.^24–26^ Thus, although there is limited evidence that phospholemman may regulate Na/K ATPase in vascular smooth muscle, the physiological relevance of this is far from certain, and its role in the control of BP has yet to be demonstrated. The primary aim of this study was, therefore, to test the hypothesis that phospholemman phosphorylation regulates vascular tone in vitro and this mechanism plays an important role in modulation of vascular function and BP in vivo.

## Methods

An expanded Methods section is available in the Data Supplement. The authors declare that all supporting data are available within the article and the Data Supplement or are available from the corresponding author on reasonable request. Access to source patient datasets is available to suitably qualified researchers through UK Biobank (UKBB) (https://www.ukbiobank.ac.uk) and GoDARTS (Genetics of Diabetes Audit and Research in Tayside; https://godarts.org).

### Animals and PLM^3SA^ Mice

All experiments were performed in accordance with the Guidance on the Operation of Animals (Scientific Procedures) Act, 1986 (UK) and the Directive of the European Parliament (2010/63/EU) and received approval by the King’s College London Ethics Review Board. Male PLM^3SA^ (phospholemman [FXYD1] in which the 3 phosphorylation sites on serines 63, 68, and 69 are mutated to alanines) knock-in mice or their wild-type (WT) littermates (13–16 weeks of age [young] or 56–60 weeks of age [old]), were used in the majority of studies. A small number of studies used male Wistar rats (250 g; Charles River, UK).

### Myography

Vascular rings were isolated from thoracic aortae of WT and PLM^3SA^ mice, or rats, mounted in a tension myograph (Danish Myo Technology) and optimally stretched. Endothelial integrity was then tested (with acetylcholine) and concentration-response curves to phenylephrine and the thromboxane A_2_ mimetic U46619 constructed in the presence or absence of ouabain. Changes in isometric tension were sampled and recorded at 100 Hz using a PowerLab and LabChart software (AD Instruments, New Zealand).

#### In Vivo Hemodynamic Assessment in Mice

Central arterial BP was measured in anesthetized mice via a pressure-tipped catheter (1.2F, Transonic) inserted retrogradely into the ascending aorta via the right common carotid artery. The left jugular vein was cannulated for phenylephrine infusion. Surface ECG was continuously recorded. Augmentation index (AI) was calculated as previously described (see also, Figure V in the Data Supplement).^27^ In separate experiments, noninvasive laser Doppler imaging (MoorLDI2 model, Moor Instruments Inc, Wilmington) was used to assess hind-limb blood flow. Continuous hemodynamic measurements in conscious mice were performed by telemetry as described previously.^28^ Cardiac structure and function were assessed by 2-dimensional echocardiography.^29^

### Immunoblotting

Thoracic aortae or mesenteric vessels were harvested from PLM^3SA^ and WT mice, cleaned of fat and connective tissue, cut into 3 or 4 pieces, and incubated at 37^°^C in gassed Krebs solution or Krebs plus phenylephrine (10 µmol/L), U46619 (1 µmol/L) or spermine NONOate (spNONO) (100 µmol/L). The tissue was snap-frozen and stored at –80°C. Tissue homogenates (4% wt/vol) were size-fractionated on SDS-PAGE gels (10% to 15%) and processed for Western blotting as previously described.^29^ α-Smooth muscle actin was used as a loading control. Signals from PLM^3SA^ samples or treated WT samples were normalized to signals from control WT samples on the same gels or, for phospholemman phosphorylation, to total phospholemman. Details of antibodies, procedures, and quantification can be found in the Data Supplement.

### Membrane Potential Measurements

Membrane potential was measured in 2-mm segments of third-order mesenteric arteries mounted in a Mulvany-Halpern wire myograph (model 400A, Danish Myo Technology, Denmark) using sharp glass microelectrodes backfilled with 2 mol/L KCl (tip resistances *≈*100 MΩ). Tension was simultaneously recorded.^30,31^

### Genomic and Phenotypic Screening in Patient Populations

We searched human genomic databases for mutations in phospholemman in the region of the phosphorylation sites. rs61753924 is a single nucleotide polymorphism (SNP) at 35 633 635 bp position (build hg19/37) on chromosome 19, in phospholemman exon 10, a C to T transition in position 1 of the codon encoding arginine 70 that generates a nonsynonymous variant with the amino acid substitution R70C. To assess the impact of this SNP on BP, we analyzed this SNP within 2 different human cohorts: UKBB^32^ and GoDARTS.^33,34^ Details of the cohorts and the analysis can be found in the Data Supplement.

The human database analysis was been conducted using the UKBB Resource under application No. 236. The genetic and phenotypic patient cohort data are available on application to UKBB (https://www.ukbiobank.ac.uk) and GoDARTS (https://godarts.org). The UKBB study has approval from the North West Multi-Center Research Ethics Committee. Any participants from UKBB or GoDARTS who withdrew consent have been removed from our analysis.

### Statistics

Data are shown as mean ± SEM. Details of the analyses used can be found in the Data Supplement.

## Results

### Prevention of Phospholemman Phosphorylation Potentiates Phenylephrine-Induced Vasoconstriction

**Figure 1.**
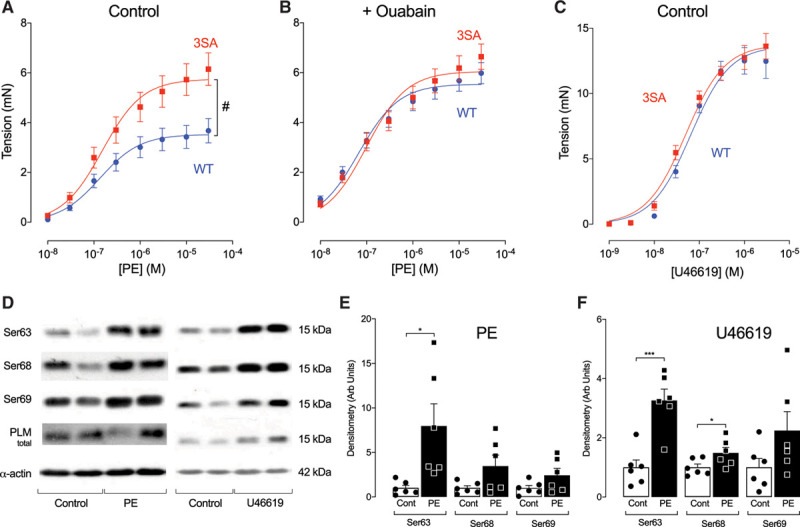
**Phospholemman (PLM) phosphorylation limits phenylephrine-induced vasoconstriction in wild-type (WT) isolated mouse aortic rings in a ouabain-sensitive manner.** Mutation of PLM to prevent phosphorylation (3SA) enhances constriction. Constriction in response to the thromboxane A2 mimetic U46619, which also increases PLM phosphorylation, was unaffected by genotype. **A**, Mutation of PLM phosphorylation sites to prevent phosphorylation markedly enhanced vasoconstriction (n=6 [WT] and n=5 [3SA], #*P*<0.001, 2-way ANOVA for comparison of maximal responses). **B**, The limitation of vasoconstriction in WT aortae was completely blocked by pretreatment with ouabain (300 μmol/L; n=5 per group). **C**, Contractile response to the thromboxane A2 mimetic U46619 was unaffected by genotype (n=6 [WT] and n=7 [3SA]). **D** through **F**, In WT aortae, both phenylephrine (10 µmol/L) and U46619 (1 µmol/L) markedly increased PLM phosphorylation (n=6 per group, *P<0.05, ***P<0.001, unpaired *t* test). Arb indicates arbitrary; Cont, control; and PE, phenylephrine.

**Figure 2.**
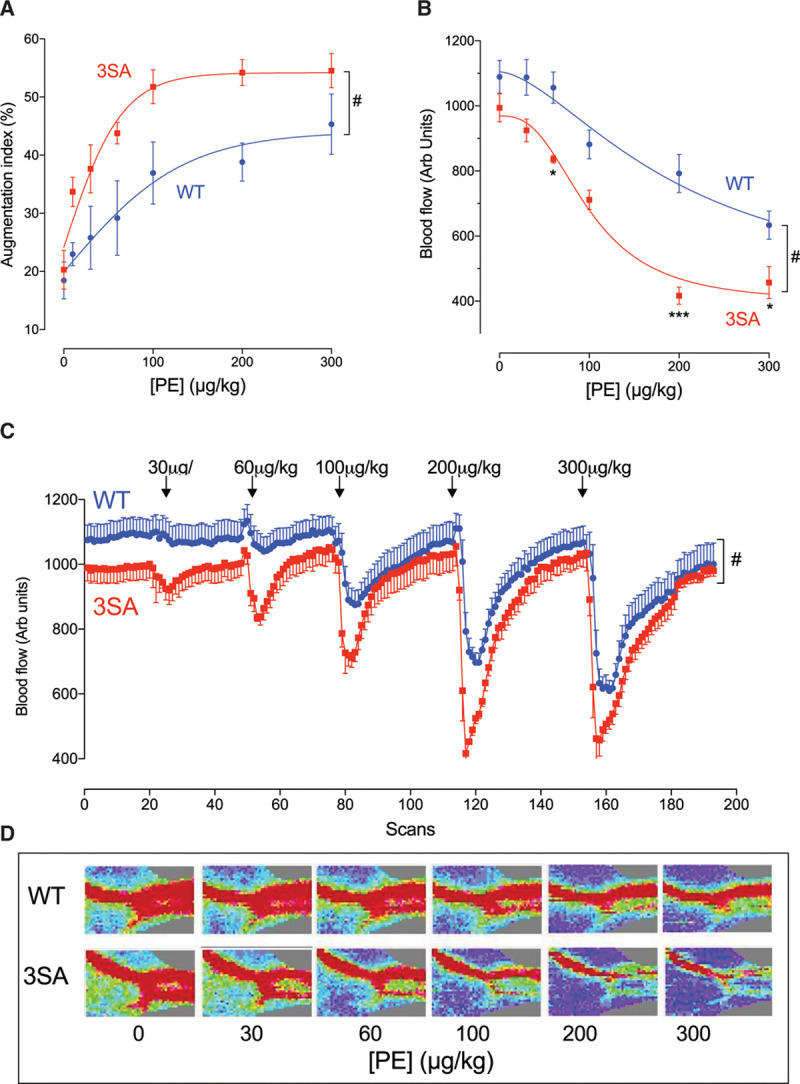
**In vivo measurements of aortic stiffness as assessed by augmentation index, and peripheral blood flow measured in anesthetized wild-type (WT) and PLM^3SA^ (3SA) mice in response to phenylephrine.** Phenylephrine (PE) was infused as a bolus injection via a left jugular vein catheter at the doses indicated. **A**, Aortic pressure measurements were made via a pressure-tipped catheter (1.2F, Transonic) introduced into the ascending thoracic aorta via the right carotid artery. Augmentation index was measured as the percentage increase in aortic pressure from the first inflection in the aortic pressure trace to the subsequent peak pressure (n=5 for both groups). **B** through **D**, In separate experiments, peripheral blood flow was measured in the hind limb by laser Doppler in response to PE bolus infusion (n=5 [3SA] and n=6 [WT]). In **B** and **C**, comparisons were made using 2-way ANOVA with Bonferroni correction and #*P*<0.001 for effect of both genotype and PE concentration and specifically *P<0.05, ***P<0.001. PLM^3SA^ indicates phospholemman (FXYD1) in which the 3 phosphorylation sites on serines 63, 68, and 69 are mutated to alanines. Arb indicates arbitrary.

Figure 1A shows the dose-dependent constriction of isolated aortic rings to phenylephrine was potentiated in tissues isolated from PLM^3SA^ mice. The response of PLM^3SA^ tissue was unaffected by ouabain, but inhibition of Na/K ATPase in WT tissue rendered the WT hyperresponsive to phenylephrine and similar to PLM^3SA^ (Figure 2B).

Although phenylephrine-induced constriction was profoundly different between genotypes, and this difference was Na/K ATPase–dependent, no differences were seen between genotypes in response to U46619 (Figure 1C). Although U46619 interestingly also induced phospholemman phosphorylation at Ser63 and Ser68 (Figure 1F), this did not limit maximal constriction in WT aortae, suggesting that, unlike phenylephrine-induced constriction, high concentrations of U46619 (0.1–30 µmol/L) mediate their constrictor effects via cellular mechanisms that are largely unaffected by phospholemman phosphorylation and Na/K ATPase activity. This is unsurprising because at these concentrations, U46619 has been shown to potently inhibit the Na/K ATPase.^35^

In parallel experiments, Western blot analysis (Figure 1D–1F) showed that phenylephrine (10 µmol/L) and U46619 (1 µmol/L) induced substantial phospholemman phosphorylation at Ser63 (phenylephrine) and Ser63 and Ser68 (U46619) in WT aortae. Expression of phospholemman, and both α1 and α2 subunits of the Na/K ATPase in aortic smooth muscle, was unaffected by genotype (Figure I in the Data Supplement).

A similar disparity between the role of Na/K ATPase in mediating the constrictor effects of phenylephrine and U46619 was also seen in rat aorta (Figure II in the Data Supplement). At concentrations of U46619 <30 nmol/L, there is a small ouabain-sensitive component (Figure IIB in the Data Supplement). Interestingly, U46619 induces phospholemman phosphorylation (Figure 1D and 1F) which, because of its concomitant Na/K ATPase inhibition, is largely without effect. This phospholemman phosphorylation is likely to reflect our previous observation that interventions raising cytosolic Ca will increase phospholemman phosphorylation by activating Ca-dependent PKCs.^22^ This may account for the small effect of ouabain on constriction in rat aorta at submaximal U46619 concentrations (ie, <30 nmol/L), where the Na/K pump may still not be fully inhibited by U46619.

On the basis of these experiments, we conclude that α_1_ adrenoceptor stimulation activates signaling pathways leading to phosphorylation of phospholemman at Ser63 (presumably through a PKC-dependent mechanism)^36^ and Na/K ATPase activation. In WT tissue, this exerts a ouabain-sensitive “relaxing” effect that limits the overall constrictor effect of phenylephrine. When phospholemman phosphorylation is prevented in PLM^3SA^ tissue, or when the Na/K ATPase is inhibited by ouabain (or U46619), this “relaxing” effect is lost, and constriction is potentiated.

#### Phospholemman-Dependent Limitation of Phenylephrine-Induced Constriction in WT Aortae Is Partially Blocked by L-NG-Nitro Arginine Methyl Ester

Previous studies have shown that endothelially derived factors modulate vascular responses to α_1_-adrenergic agonists via Na/K ATPase–dependent mechanisms.^4,15–18,37^ Furthermore, we have previously demonstrated that NO exerts phospholemman-dependent modulation of Na/K pump in cardiac tissue.^38^ We therefore hypothesized that at least part of the mechanism limiting phenylephrine-induced constriction in WT aortae may be NO-dependent. Figure IIIA in the Data Supplement shows the enhanced response of PLM^3SA^ tissues to phenylephrine (cf WT; Figure 1A). Subsequent nitric oxide synthase (NOS) inhibition with L-NG-Nitro Arginine Methyl Ester (L-NAME; 300 μmol/L) increased the dose-dependent contractile response to phenylephrine in both genotypes, but the relative potentiation was more profound in WT mice than PLM^3SA^ (Figure III in the Data Supplement). As a result of this potentiation, the difference in contractile responses between genotypes after NOS inhibition was substantially reduced but still statistically significant (Figure IIIB in the Data Supplement).

These data suggest that in WT aortae, the ouabain-sensitive mechanism limiting phenylephrine-induced constriction has 2 components: a NOS-dependent component (blocked by L-NAME) and a NOS-independent component (which persists in L-NAME).

#### Aortic Rings From WT Mice Are More Sensitive to an NO Donor

To further investigate the role of phospholemman phosphorylation in NO-dependent regulation of the Na/K pump, in the next series of experiments, we compared NO-dependent relaxation of aortic rings from both genotypes preconstricted with a low concentration of U46619. The U46619 concentration used in these experiments was 40 nmol/L because at this concentration there is still an ouabain-sensitive modulation of constriction (Figure 2B), whereas at higher concentrations, U46619 is reported to directly inhibit Na/K ATPase.^35^ Initial tension after treatment with U46619 was the same across genotypes, but spNONO-induced relaxation curves were left-shifted in aortic rings from WT mice, which reflects higher sensitivity of this genotype to NO (Figure IVA in the Data Supplement). Interestingly, pretreatment with ouabain (100 μmol/L) profoundly limited the maximal relaxation response to NO and completely abolished the shift in the K_m_ for spNONO between genotypes (Figure IVB in the Data Supplement). These results demonstrate that (1) phospholemman-dependent Na/K pump activation is one of the mechanisms involved in NO-dependent relaxation, (2) Na pump–independent mechanisms are not different between genotypes, and (3) phospholemman phosphorylation facilitates NO-dependent relaxation of WT aortic rings. Further evidence for a role of phospholemman phosphorylation in NO-dependent relaxation was seen in immunoblot experiments. Treatment of isolated aortic segments from WT mice with spNONO (100 µmol/L) time-dependently increased phospholemman phosphorylation (Figure IVC–IVE in the Data Supplement).

Thus, these in vitro experiments show that phospholemman phosphorylation and consequent Na/K ATPase activation both limit the contractile response to phenylephrine and enhance NO-dependent relaxation. Our next aim was to test whether such in vitro observations made in aortic rings are also seen in vivo and to establish whether phospholemman phosphorylation therefore plays a role in regulating vascular tone and BP.

#### Vascular Constriction in Response to Phenylephrine Is Profoundly Enhanced in PLM^3SA^ Mice In Vivo

We measured AI in anesthetized mice as an index of aortic stiffness/tone in vivo (Figure 2A). AI was measured in WT and PLM^3SA^ mice at baseline and during phenylephrine infusion (IV 10–300 μg/kg). Baseline AI was not different between genotypes, but phenylephrine treatment induced a dose-dependent elevation of AI in both genotypes. Furthermore, the increase in AI in response to phenylephrine was significantly enhanced in PLM^3SA^ compared with WT mice (Figure 2A).

Phenylephrine-induced differences in AI could reflect differences in cardiac contractility, vascular stiffness, or both. To determine the contribution of any genotype-specific cardiac response to phenylephrine infusion, in separate experiments, isolated hearts were treated with phenylephrine (Figure VC in the Data Supplement). Phenylephrine modestly increased contractility of both WT and PLM^3SA^ hearts to an identical extent. We conclude that the greater phenylephrine-induced elevation of AI in PLM^3SA^ mice reflects enhanced vascular contractility in this model.

To establish whether this enhanced vasocontractile response to phenylephrine in the PLM^3SA^ mice is unique to aorta (or is a general feature of other vascular beds), blood flow in response to phenylephrine bolus injection was measured in the hind limb. Noninvasive laser Doppler hind-limb imaging in the 2 genotypes showed no differences in blood flow under baseline conditions (Figure 2B–2D). However, acute bolus phenylephrine infusion (IV 30–300 μg/kg) induced a more profound reduction of blood flow in PLM^3SA^ mice than in WT littermates (Figure 2B–2D) at all phenylephrine concentrations. These data are consistent with the observation in Figure 2A of an enhanced constrictor response in aorta (AI) and augmented vascular contractility in phospholemman mutant mice in response to phenylephrine treatment in vivo.

#### BP Changes in PLM^3SA^ Mice: Changes in Baroreceptor Gain and Autonomic Function

**Figure 3.**
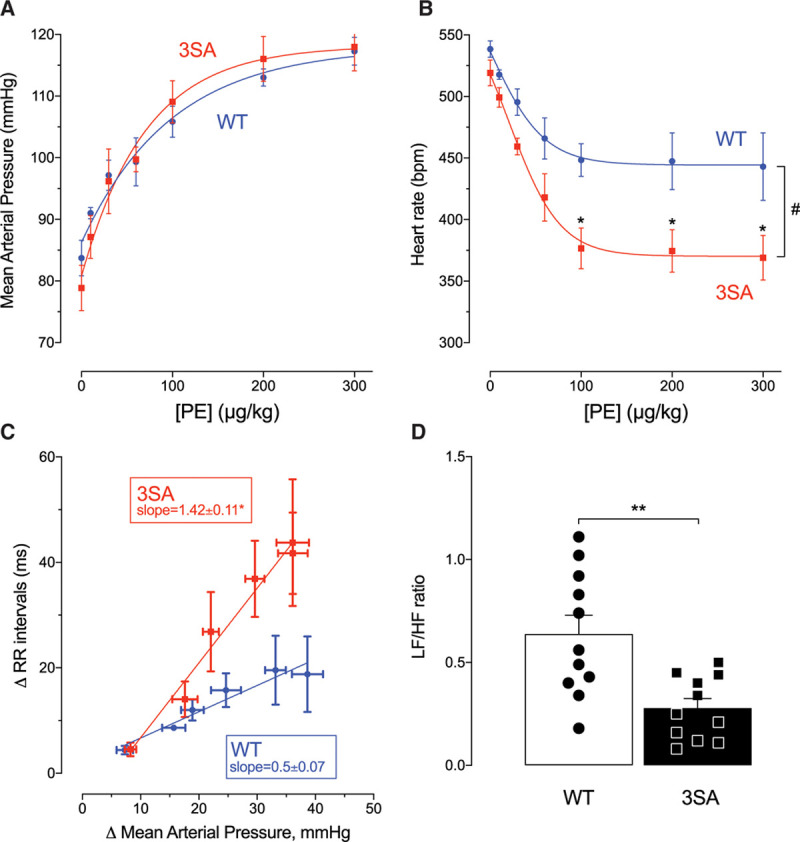
**In vivo measurements of mean arterial pressure (MAP), heart rate, baroreceptor reflex gain, and autonomic function in anesthetized wild-type (WT) and PLM^3SA^ (3SA) mice in response to phenylephrine.** Phenylephrine (PE) was infused as a bolus injection via a jugular vein catheter at the doses indicated. **A**, MAP measurements were made as described in Figure 2, and heart rate (**B**) was a calculated from the pressure trace. Baroreceptor reflex gain (BRG) was estimated by plotting the change in beat-beat interval (ΔRR) as a function of change in MAP (ΔMAP) in response PE infusion (**C**) with BRG estimated as the slope of this relationship (*P<0.05). The low/high frequency (LF/HF) component (sympathetic dominance) of heart rate variability measured in conscious telemetered mice under control conditions is shown in **D**. **A** through **C**, n= 5 per group. **D**, n=11 per group. **B**, comparisons were made using 2-way ANOVA with Bonferroni correction and #*P*<0.001 for effect of both genotype and PE concentration and specifically *P<0.05. Difference in **D** was tested using an unpaired *t* test, **P<0.005. PLM^3SA^ indicates phospholemman (FXYD1) in which the 3 phosphorylation sites on serines 63, 68, and 69 are mutated to alanines.

Baseline BP was the same in conscious and anesthetized mice of both genotypes (Figure 3A, Figure VIA in the Data Supplement). Given the profoundly enhanced constrictor response of PLM^3SA^ mouse to phenylephrine both in vitro and in vivo, surprisingly, phenylephrine infusion (IV 10–300 μg/kg) induced identical dose-dependent pressor responses in WT and PLM^3SA^ mice (Figure 3A). This appears to be explained by differences between genotypes in their baroreceptor function. Figure 3B shows that the pressure-induced bradycardia was far more profound in PLM^3SA^ than WT mice. Baroreceptor sensitivity can be estimated by plotting the change in heart rate (RR interval) as a function of change in BP (Figure 3C). From this figure, it is clear that the baroreceptor reflex gain, as measured by the slope of this relationship, is almost 3 times higher in PLM^3SA^ (1.42±0.11 ms·mm Hg^–1^) than WT mice (0.5±0.07 ms·mm Hg^–^^1^). This baroreceptor adaptation clearly allows the PLM^3SA^ mouse to maintain normal BPs at rest, and over a range of pressor interventions, despite hypercontractile vascular smooth muscle. Figure VIB and VIC in the Data Supplement also shows that these changes in baroreceptor reflex gain allow the PLM^3SA^ mice to maintain comparable changes in systolic BP in response to acute environmental stress by limiting heart rate changes. Further evidence for autonomic adaptation in these mice is shown in the heart rate variability analysis in Figure 3D and autonomic balance (Figure VID in the Data Supplement). PLM^3SA^ mice show significantly reduced low- to high-frequency ratio (Figure 3D) consistent with a chronic adaptive reduced sympathetic dominance and altered autonomic balance (Figure VID in the Data Supplement).

### Membrane Potential Measurements in Mesenteric Vessels: Role of Electrogenic Na/K Pump Activation?

**Figure 4.**
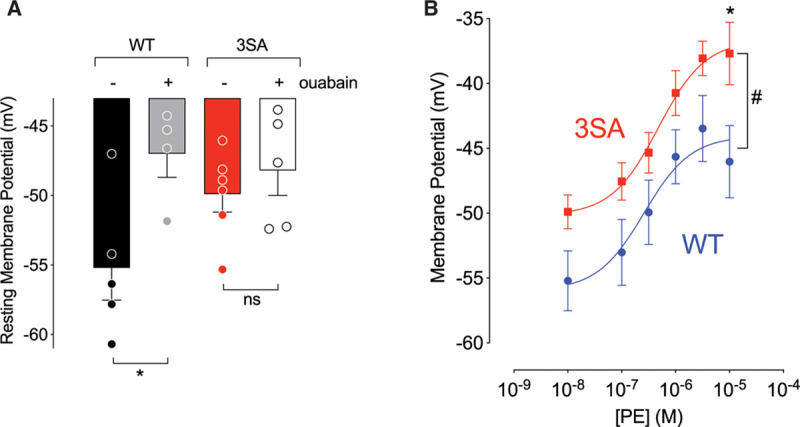
**Effect of phenylephrine (PE) on membrane potential measured in mesenteric vessels isolated from wild-type (WT) and PLM^3SA^ (3SA) mice. A**, Effect of ouabain (300 µmole/L) on basal membrane potential measured in WT and PLM^3SA^ mesentery. Resting membrane potential was more hyperpolarized in WT than PLM^3SA^ vessels—an effect that was lost when vessels were exposed to ouabain. Comparisons were made using 1-way ANOVA followed by an unpaired *t* test (*P<0.05). **B**, PE induces an equivalent dose-dependent net depolarization in both PLM^3SA^ and WT vessels with the WT vessels starting approximately 5 mV more hyperpolarized. Comparisons were made using 2-way ANOVA with Bonferroni correction and #*P*<0.0001 for effect of both genotype and PE concentration and specifically *P<0.05 (n=4 [WT] and n=6 [3SA]). PLM^3SA^ indicates phospholemman (FXYD1) in which the 3 phosphorylation sites on serines 63, 68, and 69 are mutated to alanines. ns indicates no significant difference.

The high-input impedance of vascular smooth muscle means that small changes in electrogenic Na/K pump activity can exert large effects on membrane potential (*E*_*m*_) and hence constriction. We therefore hypothesized that the ouabain-sensitive differences in response to phenylephrine are mediated by changes in *E*_*m*_. Figure 4A shows that at rest, *E*_*m*_ of WT mesenteric resistance vessels was –55±1.0 mV. Of this, about –8 mV can be attributed to the outward current carried by Na/K ATPase because ouabain, in WT vessels, reduces *E*_*m*_ to –47±0.8 mV. In the PLM^3SA^ mouse, *E*_*m*_ was considerably depolarized at rest (–50±0.5 mV), and *E*_*m*_ was no longer sensitive to ouabain—suggesting that, at rest, in the PLM^3SA^ mouse, the contribution of the electrogenic Na/K ATPase to membrane potential is minimal. In WT mesentery (as in aorta, Figure 1D and 1E), phospholemman was basally phosphorylated at Ser63 (Figure VII in the Data Supplement). However, unlike in aorta, phenylephrine did not induce further phospholemman phosphorylation at either Ser63 or Ser68 (Figure VII in the Data Supplement). The ability of phenylephrine to induce phospholemman phosphorylation in aortic but not mesenteric vessels suggests that although the Na/K ATPase and phospholemman exert tonic effects on vascular tone and membrane potential, the ability for this to be modulated by phenylephrine may vary in different vascular beds. The inability of phenylephrine to induce phospholemman phosphorylation also explains the similar constrictor responses to phenylephrine seen in WT and PLM^3SA^ mesenteric vessels (Figure VIII in the Data Supplement).

### Role of Phospholemman Dephosphorylation in Aging-Induced Essential Hypertension in Mice

**Figure 5.**
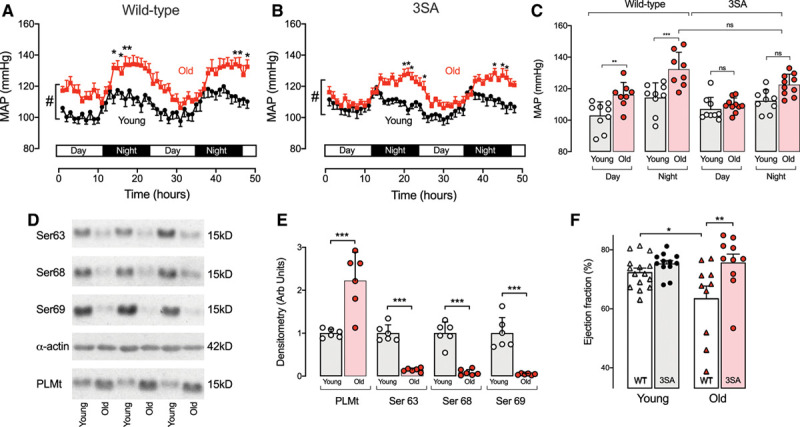
**Aging-induced essential hypertension was associated with significant PLM hypo-phosphorylation in wild-type mice.** PLM^3SA^ mice were substantially protected against both this aging-induced hypertension and the associated cardiac hypertrophy and contractile dysfunction. **A**, Aging-induced essential hypertension measured over 48 hours in telemetered young (14–16 weeks) and old (57–60 weeks) wild-type (WT) mice. **B**, In PLM^3SA^ mice, the diurnal fluctuation of BP was similar to that in young WT mice, but nocturnal hypertension was significantly attenuated in PLM^3SA^ mice. **C**, Average mean arterial pressures (MAPs) recorded in young and old mice during day and night by genotype. **D** and **E**, In WT mice, PLM was substantially hypophosphorylated at Ser63, Ser68, and Ser69 in aortic tissue from old mice (n=6 per group, *P<0.05). **F**, PLM^3SA^ mice were also protected against aging-induced cardiac contractile dysfunction (ejection fraction; see also Figure XI in the Data Supplement). **A** and **B**, Comparisons were made using 2-way ANOVA with Bonferroni correction and #*P*<0.001 for effect of both age and time and specifically *P<0.05. **C** and **F**, Comparisons were made using 1-way ANOVA with Bonferroni correction. **E**, Unpaired *t* test was used for comparison. n=10 (adult, WT), n=9 (old, WT), n=11 (adult, PLM^3SA^), n=11 (old, PLM^3SA^), *P<0.05, **P<0.01, ***P<0.001. 3SA indicates PLM3SA; PLM, phospholemman; and PLM^3SA^, phospholemman (FXYD1) in which the 3 phosphorylation sites on serines 63, 68, and 69 are mutated to alanines. Arb indicates arbitrary; ns, no significant difference; and PLMt, total phospholemman.

Having demonstrated the importance of phospholemman phosphorylation in the regulation of vascular smooth muscle tone and BP in young mice, we investigated whether changes in phospholemman phosphorylation are associated with aging-induced essential hypertension. Figure 5A shows the diurnal variation in mean arterial pressure in young mice (14–16 weeks of age) and old hypertensive mice (57–60 weeks of age). In aortae from young mice, phospholemman was significantly basally phosphorylated at all 3 serine residues (Figure 5D and 5E). However, in old hypertensive mice, phospholemman was substantially hypophosphorylated and overexpressed (Figure 5D and 5E). There were no significant differences in α1 or α2 Na/K ATPase subunit expression with age (Figure IXA in the Data Supplement), but the increase in phospholemman expression resulted in a significant relative excess of unphosphorylated phospholemman in old mice (Figure IXB in the Data Supplement).

BP in young and old WT and PLM^3SA^ mice is shown in Figure 5B and 5C. Whereas WT mice show substantial aging-induced hypertension during both the day and night, PLM^3SA^ mice are completely protected against hypertension at rest (day) and substantially protected against hypertension during activity (night). In old WT mice, average mean arterial pressure at rest (day) was +13 mm Hg higher than in young mice, and this hypertension was completely prevented in PLM^3SA^ mice. During activity (night), old WT mice showed a substantial aging-induced hypertension (+18 mm Hg) that was substantially abrogated in the PLM^3SA^ genotype (+10 mm Hg). The heart rate variability differences seen in young mice (Figure 3D) were still present in aged mice (Figure X in the Data Supplement). PLM^3SA^ mice were protected not only against aging-induced hypertension but also against aging-induced cardiac dysfunction (Figure 5F, Figure XI in the Data Supplement).

### Homologous Variant in a Human Population Influences BP

**Figure 6.**
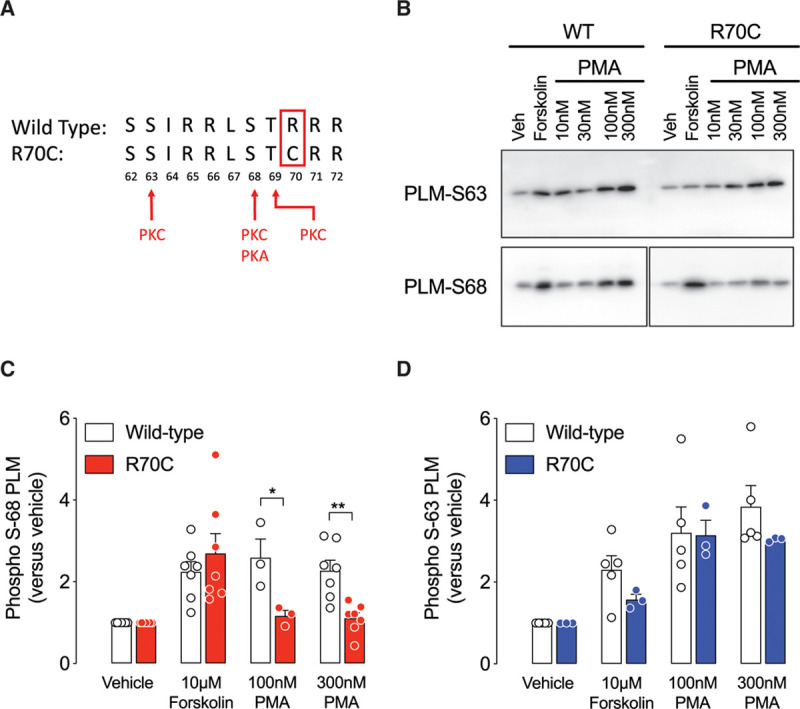
**The R70C mutation limits protein kinase C (PKC) but not protein kinase A (PKA) phosphorylation of phospholemman (PLM) at Ser68 but does not affect phosphorylation at the more remote Ser63. A**, Position of the R70C mutation in human PLM. The PKC and PKA phosphorylation sites at Ser63, Ser68, and T69 are indicated. **B**, Phosphospecific immunoblots after activation of PKA or PKC in transfected wild-type (WT) and R70C human embryonic kidney cells. **C** and **D**, Quantified PLM Ser68 and Ser63 phosphorylation, respectively. Unpaired *t* test was used for comparison (**C**, n=7 per group and **D**, n=3–5 per group). *P<0.05; **P<0.005. All other comparisons between WT and R70C not significant. PMA indicates Phorbol 12-myristate 13-acetate; and Veh, vehicle.

We identified an SNP in phospholemman that generates an R70C amino acid substitution in phospholemman. We hypothesized that R70C is unlikely to influence phosphorylation at phospholemman Ser68 by protein kinase A (consensus motif RRX*S/T*). PKC isoforms generally require positive charges in positions +3, +2, –2, and –3 (consensus RRX*S/T*XRR),^39^ so we hypothesized that phosphorylation by PKC at Ser68 (but not Ser63 and Thr69) may be perturbed (Figure 6A). We also investigated the possibility that mutation R70C destroys the proposed endoplasmic reticulum retention motif in phospholemman.^40^ In human embryonic kidney cells, the R70C phospholemman mutation is not phosphorylated at Ser68 by PKC (Figure 6B and 6C), and displays enhanced cell surface localization and palmitoylation (Figure XII in the Data Supplement), whereas phosphorylation at the more remote Ser63 site by PKC was unaffected by the R70C mutation (Figure 6D).

To assess the impact of this SNP, we analyzed data from UKBB including a total of 357 151 unrelated individuals of European ancestry, with 159 204 men and 197 947 women (Table I in the Data Supplement). In total, 7114 individuals carried at least 1 copy of the rare T allele of the SNP rs61753924, with a minor allele frequency of 1% and no evidence of departure from Hardy-Weinberg equilibrium (*P*=0.33). At recruitment, mean age was 56.4 years, mean systolic BP (SBP) was 140.5 mm Hg, and mean diastolic BP (DBP) was 84.1 mm Hg, with 50.2% of individuals being hypertensive (SBP≥140 mm Hg or DBP≥90 mm Hg), and 17.2% reported they were taking BP-lowering medication.

**Figure 7.**
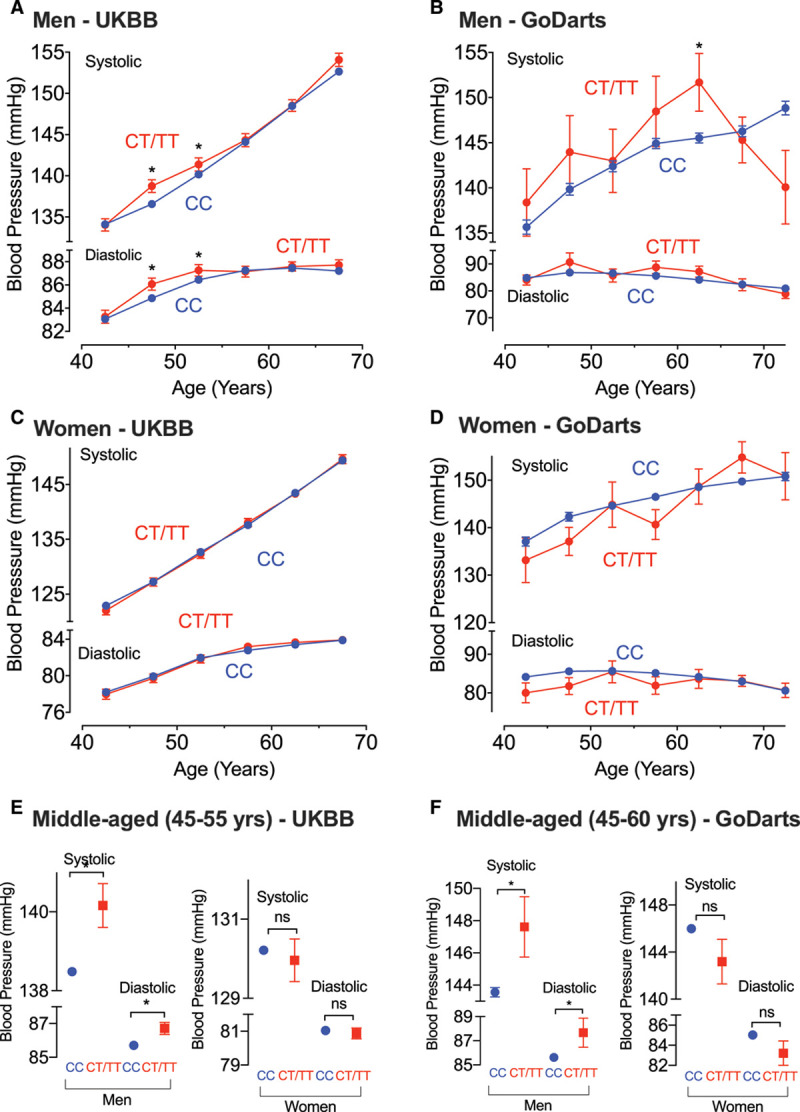
**The R70C phospholemman mutation (rs61753924) is associated with an age- and sex-dependent effect on BP in 2 human cohorts—UK Biobank (UKBB) and GoDARTS.** CC is the comparator (control), and CT and TT represent heterozygote and homozygote genotypes. Mean systolic and diastolic blood pressures (BPs), within 5-year age intervals, are shown in both cohorts. The T-allele was associated with a significant increase in both systolic and diastolic BP in men (**A** and **B**) but not women (**C** and **D**), and was especially associated within middle-aged men (**E** and **F**). **A** through **D**, Plot of the mean BP estimates within each age group and tests for significant differences between consecutive age groups, according to nonoverlapping 95% CIs. **E** and **F**, Mean BP estimates within middle-aged subjects corresponding to results from the age-stratified linear regression analyses, testing for a significant difference in mean BP between CC vs CT/TT genotype groups. Sample sizes: UKBB, n=159 204 men (**A**), 197 947 women (**C**), 44 995 middle-aged (45–55 y) men, and 58 194 middle-aged women (**E**). GoDARTS: n=4460 men (**B**), 3324 women (**D**), 1786 middle-aged (45–60 y) men, and 1187 middle-aged women (**F**). *P<0.05. GoDARTS indicates Genetics of Diabetes Audit and Research in Tayside. ns indicates no significant difference.

Within UKBB, this SNP is associated with BP in a sex- and age-dependent manner (Figure 7). The rare T allele is significantly associated with increasing levels of both SBP (0.77 mm Hg [95% CI, 0.14–1.4]; *P*=0.017) and DBP (0.4 mm Hg [95% CI, 0.02–0.79]; *P*=0.04) in men. However, there is no significant association with BP in either the overall sample (*P*=0.16 for SBP; *P*=0.11 for DBP) or within women (*P*=0.85 for SBP; *P*=0.75 for DBP). This suggests a sex-specific association. Focusing on results within men, Figure 7A illustrates an age-dependent effect of this SNP, with the association significant only in the age-stratified analyses for men 45 to 50 years and 50 to 55 years old. Indeed, an initial age-stratified analysis comparing men ≤55 years old versus men >55 years old shows that the rare T allele is significantly associated with increasing levels of both SBP (1.2 mm Hg [95% CI, 0.30–2.11]; *P*=8.89×10^–3^) and DBP (0.79 mm Hg [95% CI, 0.18–1.4]; *P*=0.01) in men <55 years old, but is not significantly associated with BP in men >55 years old (*P*=0.28 for SBP; *P*=0.48 for DBP). We therefore conclude that the SNP has strongest effect in middle-aged men, with the rare T allele significantly increasing both SBP (1.7 mm Hg [95% CI, 0.59–2.8]; *P*=2.62×10^–^^3^) and DBP (1.02 mm Hg [95% CI, 0.29–1.74]; *P*=5.96×10^–^^3^) in men 45 to 55 years of age (Figure 7E).

In a similar analysis in the GoDARTS cohort (n=7784), the proportion of individuals carrying the T allele was slightly higher at 3% compared with UKBB, although in Hardy-Weinberg equilibrium (*P*=0.7). We replicated the findings from UKBB with the T allele showing significant association with SBP increased by 4.5 mm Hg (95% CI, 0.72 to 8.27; *P*=0.02) only in middle-aged men 40 to 65 years.

## Discussion

**Figure 8.**
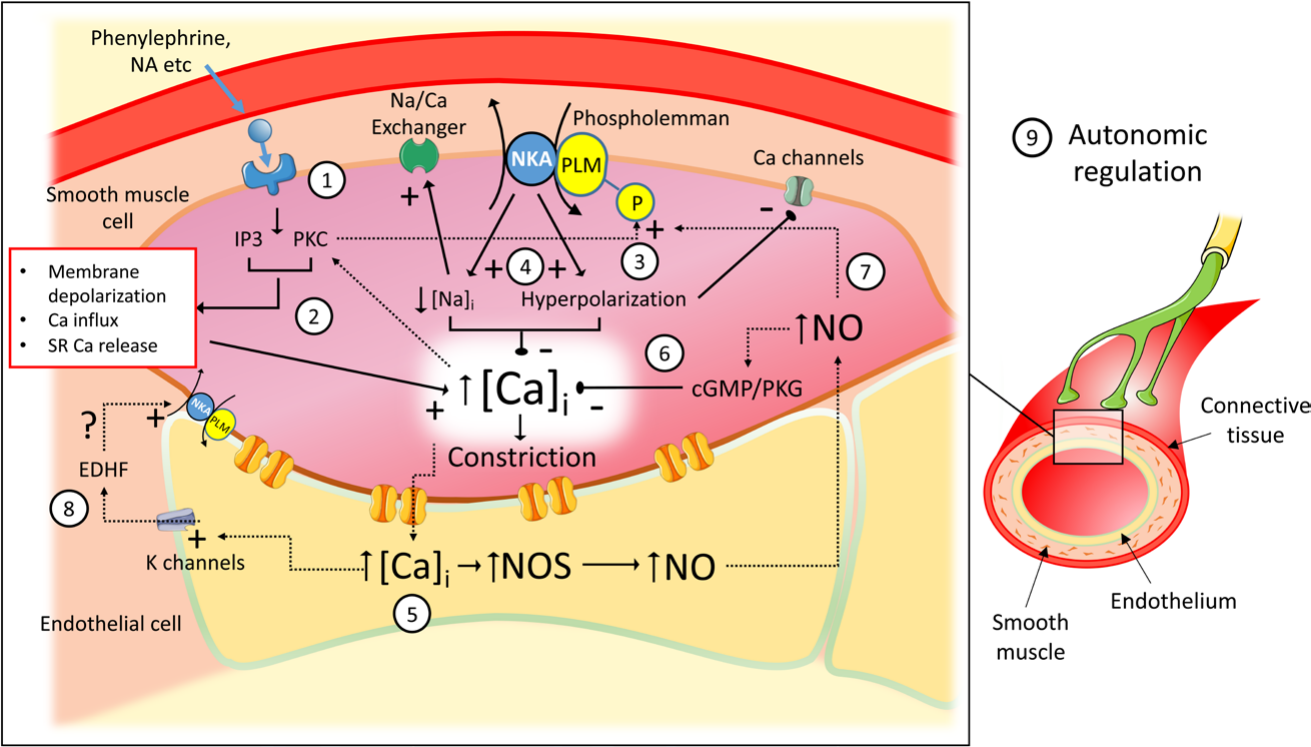
**Proposed mechanisms by which phospholemman (PLM) phosphorylation and Na/K ATPase activation modulate vascular contractility. 1**, Activation of cell surface receptors (ie, by phenylephrine or noradrenaline [NA]) activates IP3 and protein kinase C (PKC). **2**, This elevates cytosolic Ca through a combination of membrane depolarization and enhanced Ca entry (principally via voltage-gated channels) and cyclic sarcoplasmic reticulum (SR) Ca release leading to constriction. **3**, PKC, whose activation is enhanced by elevation of cytosolic Ca, phosphorylates phospholemman (at either Ser63 or both Ser63 and Ser68). **4**, Phosphorylated PLM disinhibits the Na/K ATPase (NKA), which generates an outward current limiting the depolarization of the cell membrane and hence limiting Ca influx through voltage-gated channels and/or lowers cytoplasmic Na facilitating Ca efflux via Na/Ca exchange. This limits the rise in Ca in the cytosol and hence limits constriction. **5**, The receptor-mediated rise in cytosolic Ca also passes to adjacent endothelial cells, where it activates both nitric oxide synthase (NOS) and the release of endothelium-derived hyperpolarizing factor (EDHF). Nitric oxide (NO) diffuses to smooth muscle cells, where it limits constriction by 2 pathways: (**i**) via the canonical cGMP/protein kinase G (PKG) pathway (**6**) and (**ii**) via activation of a PLM/NKA-dependent pathway (**7**). EDHF release from endothelial cells may also limit depolarization, possibly via activation of ouabain-sensitive NKA. **8**, Basal phosphorylation of PLM influences vascular tone via these mechanisms and, in the PLM^3SA^ mouse, adaptive changes in baroreceptor reflex gain and autonomic control maintain baseline resting blood pressure despite enhanced vascular reactivity (**9**). In aged mice, and in humans expressing the R70C mutation, defects in this PLM/NKA mechanism contribute to hypertension. PLM^3SA^ indicates phospholemman (FXYD1) in which the 3 phosphorylation sites on serines 63, 68, and 69 are mutated to alanines.

These studies demonstrate the importance of phospholemman-mediated regulation of vascular smooth muscle Na/K ATPase activity, membrane potential, tone, and BP. Phospholemman phosphorylation stimulates Na/K ATPase, which limits agonist-induced vascular smooth muscle depolarization and hence limits constriction (Figure 8). The presence of an SNP that abolishes phospholemman phosphorylation at Ser68 by PKC is significantly associated with increased BP in middle-aged men. Mutation of the phosphoregulatory sites on phospholemman in mice blocks this pathway and renders vascular smooth muscle hyperresponsive to phenylephrine. These PLM^3SA^ mice show a chronic adaptation of their baroreceptor sensitivity, which can maintain normal BP at rest in response to exogenous phenylephrine (or environmental stress) despite a profoundly enhanced peripheral vasoconstriction. The autonomic adaptations in these mice can prevent chronic essential hypertension induced by aging. In aged WT mice, the combination of increased phospholemman expression and profound phospholemman hypophosphorylation is associated with hypertension, which was largely abrogated by autonomic adaptations in PLM^3SA^ mice. This raises the possibility that phospholemman and the prevention of its dephosphorylation and Na/K ATPase inhibition may provide a novel therapeutic target in aging-induced essential hypertension.

### Mechanism of Phospholemman-Mediated Modulation of Vasoconstriction

Many studies have shown that Na/K ATPase modulates vascular tone independent of changes in intracellular Na.^3,41^,^42^ We therefore propose that activation of vascular smooth muscle Na/K ATPase, and the phospholemman-mediated ouabain-sensitive relaxing effect, involve increased electrogenic Na/K pump current, which hyperpolarizes the cell membrane or limits agonist-induced depolarization. Preventing phospholemman phosphorylation in PLM^3SA^ mice profoundly depolarizes resting membrane potential in mesenteric arteries from –55±2 mV (WT) to –50±1 mV (PLM^3SA^). The steep current-voltage relationship of the L-type Ca channel window current means that such a change in membrane potential can significantly increase steady-state Ca influx and hence increase vascular tone in PLM^3SA^ vessels. In WT vessels, this ouabain-sensitive 5 mV hyperpolarization persisted across the entire range of depolarizations induced by phenylephrine. In these mesenteric vessels, this hyperpolarization was not further enhanced by a concentration-dependent phenylephrine-induced Na/K pump activation, because phospholemman phosphorylation was not further increased by phenylephrine treatment (unlike in aorta).

### Differences in the Role of Phospholemman in U46619 and Phenylephrine-Induced Constriction

Our data suggest that the constrictive effects of phenylephrine are limited by 2 factors: (1) phospholemman phosphorylation activating the Na/K ATPase and (2) endothelially derived NO release. Neither of these occurs in U46619 constriction because the Na/K ATPase is already inhibited by U46619, and U46619 has been reported to *inhibit* rather than promote NO release from the endothelium.^43,44^

### Role of NO in Phospholemman Modulation of Relaxation

Figure IV in the Data Supplement also shows that NO, as in cardiac muscle,^38^ can also induce phospholemman phosphorylation. Hence, a component of classical NO-mediated relaxation is likely to involve the activation of Na/K ATPase via this mechanism. Because phenylephrine also stimulates the generation of NO,^37^ it can therefore limit constriction through direct PKC-mediated phosphorylation of phospholemman and also via more classical NO-dependent downstream pathways that are phospholemman-independent.

NOS inhibition significantly augmented dose-dependent constriction to phenylephrine in both genotypes (Figure III in the Data Supplement), demonstrating the presence of a phospholemman-independent effect of phenylephrine that antagonizes constriction. However, potentiation of constriction by L-NAME was most profound in the WT aortic segments (Figure III in the Data Supplement), suggesting that phospholemman phosphorylation plays a role in NO-dependent modulation of phenylephrine-induced constriction. Interestingly, even in the presence of L-NAME, aortic constriction to phenylephrine was higher in PLM^3SA^ mice than in WT (Figure III in the Data Supplement), demonstrating that phenylephrine activates a complex array of NO-dependent/independent and phospholemman-dependent/independent mechanisms that antagonize phenylephrine-induced constriction.

The assumption is that the PLM^3SA^ mutation in PLM mediates its effects by influencing Na/K ATPase activity in vascular smooth muscle. However, the PLM^3SA^ is a global knock-in under the control of the endogenous phospholemman promotor. However, FXYD proteins are ubiquitous, and it is likely that vascular endothelium will express its own FXYD protein, which has yet to be identified. It seems unlikely that this will be FXYD1 (phospholemman), as all reports suggest that this is muscle-specific. We have failed to detect phospholemman in endothelial cell cultures (human umbilical vein endothelial cells) (data not shown). None of the other members of the FXYD family contain the cytoplasmic tail with the regulatory phosphorylation site, so they could not be candidates for modulation by phosphorylation. Should aortic vascular endothelium express FXYD1 (phospholemman), this could affect the Na gradient *in endothelial cells* and hence intracellular Ca and NOS-induced NO release.

### In Vivo Adaptations and Control of BP

The role of phospholemman phosphorylation in the regulation of vascular tone was also confirmed in vivo. AI is influenced by the velocity and amplitude of the aortic pressure wave reflection and is proportional to arterial stiffness.^45–48^ In PLM^3SA^ mice, AI was similar to that in WT at baseline, but the response to acute administration of phenylephrine was profoundly amplified in PLM^3SA^ mice (Figure 2A). In addition, hind-limb resistance vessels also show enhanced constriction in response to bolus infusion of phenylephrine in PLM^3SA^ mice (Figure 2B–2D).

Surprising, despite this profoundly enhanced vascular responsiveness, in vivo BP was not different between the genotypes even during phenylephrine infusion (Figure 3A). This lack of enhanced pressor response to phenylephrine despite a profound increase in both central and peripheral vasoconstriction is explained by an enhanced reflex bradycardia (Figure 3B). This is best demonstrated by the change in baroreflex gain in Figure 3C. The PLM^3SA^ mouse has a baroreflex sensitivity almost 3 times that of the WT mouse (1.42±0.11 versus 0.5±0.07 ms·mm Hg^–1^). There also appears to be an autonomic compensation under baseline conditions that is reflected in reduced the low/high frequency ratio (sympathetic dominance) of heart rate variability (Figure 3D) measured in conscious telemetered mice. Pharmacological inhibition of sympathetic and parasympathetic activity revealed substantially lower activity in both limbs of the autonomic nervous system and lower intrinsic heart rate in PLM^3SA^ mice (Figure VID in the Data Supplement). New environment stress induced similar elevation of BP in both genotypes, but again, the chronotropic response was limited in PLM^3SA^ mice (Figure VIB and VIC in the Data Supplement). Thus, different autonomic control of heart rate, lower intrinsic pacemaker activity, and higher baroreflex sensitivity appear to underlie adaptive mechanisms allowing PLM^3SA^ mice to maintain normal arterial BP despite the absence of phospholemman-dependent modulation of vascular function.

### Phospholemman Phosphorylation and Aging-Induced Essential Hypertension

Aging in WT mice is associated with essential hypertension (Figure 5A and 5C) and a gradual increase in arterial stiffness.^49^ There is a profound age-related reduction of phospholemman phosphorylation at all phospholemman phosphorylation sites accompanied by increased phospholemman expression in aortic tissue (Figure 5D and 5E). This combination of increased phospholemman expression and profound dephosphorylation will inhibit Na/K ATPase. A reduction in Na/K ATPase activity may not only affect vascular tone, as described in this study, but also lead to chronic vascular remodeling.^7^ PLM^3SA^ mice were significantly protected against this aging-induced hypertension, and heart rate variability analysis suggests that these old PLM^3SA^ mice maintain their adaptive changes in autonomic function in a way that protects them against aging-induced hypertension (Figure X in the Data Supplement).

Our previous studies demonstrated that PLM^3SA^ mice are more prone to cardiac hypertrophy and dysfunction in a banding model of heart failure.^29^ However, it is interesting to note that aged PLM^3SA^ mice in this study show no cardiac hypertrophy and reduced contractile dysfunction compared with aged WT littermates (Figure 5F, Figure XI in the Data Supplement). This suggests that the reduction in the trigger for hypertrophy (ie, essential hypertension) in aging PLM^3SA^ mice can compensate for the previously shown vulnerability of these mice to pressure overload.

Although it is tempting to suggest that the downregulation of Na/K pump function in WT mice is causally associated with aging-induced hypertension, and the protection afforded in the PLM^3SA^ mouse suggests a causal role for phospholemman phosphorylation, it is also possible that the chronic downregulation of sympathetic outflow in the PLM^3SA^ mouse is akin to a lifetime of β-blockade, and the changes in WT phospholemman are merely coincidental.

### Therapeutic Potential of Targeting the Phospholemman/NKA Regulatory Axis in Hypertension

In humans, the R70C phospholemman mutation (rs61753924) has a significant impact on BP profiles. At middle age, possession of the T allele is associated with both increased SBP and DBP levels. In particular, middle-aged male carriers have 1.7 mm Hg higher mean SBP. Within the general population, an effect of this size for a single SNP alone is substantial. The results of BP genetic association studies, considering the effect of all common variants (minor allele frequency≥1%) associated with BP, show that the maximum effect size for a single common variant is ≈1.2 mm Hg for SBP, with the average effect size across all SBP-associated SNPs being ≈0.3 mm Hg.^50^ Furthermore, even the genetic risk score combining the effects of all 901 independent BP-associated SNPs reported in 2018 shows an aggregated risk of only ≈10 mm Hg difference in mean SBP when comparing individuals in the top versus bottom 20% of the genetic risk distribution. Of course, there is an increasing trend between the effect size of associated SNPs and their minor allele frequency, with only rare variants expected to have larger effects, and indeed, this is a low-frequency SNP with minor allele frequency=1%; however, even in recent BP genetic studies investigating rare variants, few individual SNPs have still been identified with an effect size for SBP of >2 mm Hg.^51^ We note, however, that because of the rare frequency of this SNP, and considering the highly polygenic nature of BP within the general human population, we do not claim any strong clinical impact for this SNP alone. Instead, it is only really genetic mutations for monogenic hypertension syndromes that are expected to have larger, clinically meaningful effect sizes for single mutation variants.^50,51^ Therefore, we suggest that the large effect size of this SNP for increasing BP levels within middle-aged men demonstrates the importance of the phospholemman/Na/K ATPase regulatory pathway in the overall control of BP. Phospholemman-mediated Na/K ATPase activation may offer therapeutic potential for poorly managed essential hypertension.

## Acknowledgments

The UKBB analysis was enabled using the computing resources of the Medical Research Council eMedLab Medical Bioinformatics Infrastructure at the National Institute for Health Research Biomedical Research Center at Barts and The London School of Medicine and Dentistry.

## Sources of Funding

This work was supported by grants from the British Heart Foundation (RG/12/4/29426 to M.J.S. and W.F. and RG/17/15/33106 to W.F.) and British Heart Foundation Center of Research Excellence awards (RE/18/2/34213, King’s College London, and RE/18/6/34217 University of Glasgow). O.R. was funded by a British Heart Foundation Intermediate Fellowship (FS/14/57/31138) and King’s College London British Heart Foundation Centre of Research Excellence Award (RE/18/2/3421). H.R.W. was funded by the National Institute for Health Research as part of the portfolio of translational research of the National Institute for Health Research Biomedical Research Center at Barts and The London School of Medicine and Dentistry, and the Medical Research Council eMedLab Medical Bioinformatics Infrastructure is supported by the Medical Research Council (MR/L016311/1).

## Disclosures

None.

## Supplemental Materials

Expanded Methods

Data Supplement Figures I–XII

Data Supplement Table I

References 52–54

## Supplementary Material


