# Supplementary material for: Phospholemman Phosphorylation Regulates Vascular Tone, Blood Pressure, and Hypertension in Mice and Humans
Source: Circulation. 2020 Dec 18;143(11):1123–38. doi: 10.1161/CIRCULATIONAHA.119.040557 (PMC7969167; doi:10.1161/CIRCULATIONAHA.119.040557)
Supplement: Supplementary file 1 [file cir-143-1123-s001.pdf]

SUPPLEMENTAL MATERIAL

Boguslavskyi *et al*

EXPANDED METHODS

Animals and PLM^{3SA} mice

All experiments were performed in accordance with the Guidance on the Operation of Animals (Scientific Procedures) Act, 1986 (UK), the Directive of the European Parliament (2010/63/EU) and received approval by King's College London Ethics Review Board. Male PLM^{3SA} knock-in mice or their WT littermates (usually 13-16 weeks old – see below), were used in the majority of studies. As described previously, PLM^{3SA} mice globally express (ie heart, smooth muscle, skeletal muscle etc) an unphosphorylatable form of PLM in which serines 63, 68 and 69 have all been mutated to alanines.[29] Heterozygote breeders were used to generate homozygote PLM^{3SA} mice and they were compared to WT littermates. In studies of ageing, mice (WT and PLM^{3SA}) were housed with free access to standard lab diet and water for either 13-16 weeks (young) or 56-60 weeks (old). All animals were subjected to a 12h/12h light/dark cycle. Male Wistar rats (250g) were purchased from Charles River, UK.

Myography

Vascular rings were isolated from the thoracic aorta of WT and PLM^{3SA} mice, or rats, and bathed in Krebs solution containing (in mmol/L) $CaCl_2$ 2.5, NaCl 118, $NaHCO_3$ 25, KCl 4.7, KH_2PO_4 1.18, $MgSO_4$ 1.16 and glucose 11, pH 7.4) maintained at 37°C and gassed with 95% O_2:5% CO_2. Aortic rings were mounted in a tension myograph (Danish Myo Technology) and optimally stretched using the DMT Normalization Module. After 30min equilibration, rings were pre-contracted using 60mmol/L KCl for 20 min and then rinsed 3 times. Endothelial integrity was tested with acetylcholine (ACh, 10µmol/L) in segments previously contracted with α_1-adrenergic agonist phenylephrine (PE, 10µmol/L). Relaxation equal to or greater than 60% was considered indicative of endothelial integrity. After 15min washout, concentration-response curves to PE and the thromboxane A_2

mimetic U46619 were constructed in the presence or absence of ouabain. In these experiments aortic rings were pre-incubated for 30min with 300µmol/L ouabain before treatment with increasing doses of PE or U46619 in ouabain-containing solution. In further experiments, PE responsiveness was investigated after 30min preincubation with the nitric oxide synthase (NOS) inhibitor L-NAME (300 µmol/L). The effect of PLM phosphorylation on NO-dependent relaxation was studied in 40nmol/L U46619-precontracted vessels treated with spermine NONOate (spNONO, 10nmol/L - 100µmol/L) in the presence or absence of ouabain (100µmol/L). Changes in isometric tension were sampled and recorded at 100Hz using a PowerLab and analysed using LabChart software (AD Instruments, New Zealand).

In vivo hemodynamic assessment in anaesthetized mice:

Mice were anesthetized by inhalation of isoflurane/O_2 mixture (2/98%). Adequacy of anesthesia was controlled by monitoring corneal reflex and respiration (rate, depth and pattern of breathing). Body temperature and heart rate were maintained within the physiological range. A pressure tipped catheter (1.2F, Transonic) was inserted retrogradely into the ascending aorta via the right common carotid artery, and the left jugular vein was cannulated for PE infusion. Surface ECG was continuously recorded via limb-lead II. After stabilization (10min), BP and ECG were recorded for 5min (baseline) and for another 60min during PE infusion (0-300µg/kg). Augmentation index (AI) was calculated as previously described by Reddy *et al*.[27] In separate experiments, non-invasive laser Doppler imaging (MoorLDI2 model; Moor Instruments Inc., Wilmington, USA) was used to assess hind-limb blood flow at baseline and during PE infusion. Mice were prepared as before (but without carotid catheterization) and the inner side of right hind-limb was shaved and blood flow measured in the same 1cm^2 segment in all animals.

In vivo hemodynamic assessment in conscious mice:

Continuous hemodynamic measurements in conscious mice were performed by telemetry as described previously.[28] Briefly, mice were anesthetized with isoflurane/O_2 mixture (2/98%) and a TA11PA-C10 probe catheter (Data Science International) was implanted into aortic arch via the left common carotid artery. Following 10 days recovery, mice were placed above the telemetric receivers and the hemodynamic parameters recorded by scheduled sampling.

In vivo assessment of cardiac structure and function

Cardiac structure and function were assessed by 2D echocardiography as previously described.[29] Briefly, mice were anesthetized (as above) and imaged using a high-resolution ultrasound system Vevo 770 (FUJIFILM VisualSonics) with a 30MHz linear signal transducer. Left ventricular dimensions and function were measured in parasternal long and short axis, aortic flow was measured with pulse-wave Doppler. Body temperature, heart rate and ECG were controlled during imaging and all experiments were performed under physiological conditions.

Immunoblotting:

Thoracic aortae were harvested from PLM^{3SA} and WT mice, cleaned of fat and connective tissue, cut into 3-4 pieces and incubated at 37°C in gassed Krebs solution or Krebs plus PE (10µmol/L), U46619 (1µmol/L) or spNONO (100µmol/L). The tissue was then snap frozen and stored at -80°C. Aortic homogenates (4%w/v) were size-fractionated on SDS-PAGE gels (10-15%). Proteins were transferred to PVDF membranes (0.45µm; GE Healthcare, UK). Immunoblots were blocked with 5% non-fat milk in PBS-Tween overnight at 4°C. Blots then were incubated 1-1.5 hours at room temperature with a primary antibody: Na/K ATPase α-1 (1:5000; Millipore) and α-2 (1:1000; Millipore), anti-FXYD1 antibody (total PLM antibody, 1:2000; Abcam), custom made anti-PLM phospho antibodies[22] and anti-actin (smooth muscle alpha isoform, 1:15000; Abcam). The anti-PLM phospho antibodies used in this investigation do not detect any PLM phosphorylation in PLM^{3SA}

mice.[29,52] After incubation with HRP-labeled secondary antibodies, blots were developed using enhanced chemiluminescence (Amersham Pharmacia Biotech). Alpha smooth muscle actin was used as a loading control. Signals from PLM^{3SA} samples or treated WT samples were normalized to signals from control WT samples on the same gels.

Membrane potential measurements:

Third-order mesenteric arteries (external diameter between 135-229µm at 70mmHg) were dissected free of adherent tissue in WT and PLM^{3SA} mice. A small artery segment (2mm) was removed and mounted in a Mulvany-Halpern wire myograph (model 400A, Danish Myo Technology, Denmark). The solution temperature was raised to 37°C, and the artery normalized to a resting tension equivalent to 90% diameter of the vessel at 70mmHg. Artery reactivity was assessed by pre-constriction to phenylephrine (PE, 0.5-3µmol/L) followed by endothelium-dependent relaxation to acetylcholine (ACh, 0.1 and 1µmol/L). Only vessels that relaxed by more than 95% were used. Vascular smooth muscle membrane potential was measured using sharp glass microelectrodes backfilled with 2mol/L KCl (tip resistances ~100MΩ), simultaneously with change in tension, as previously described.[30,31] Membrane potential was recorded via a pre-amplifier (Neurolog, Digitimer Ltd, UK) and sampled and recorded at 100Hz using a PowerLab (AD Instruments, New Zealand). All drugs were added directly to the bath.

Genomic and phenotypic screening in patient populations

We searched human genomic databases for mutations in PLM in the region of the phosphorylation sites. rs61753924 is a single nucleotide polymorphism (SNP) at 35,633,635 base pairs position (build hg19/37) on chromosome 19, in PLM exon 10, a C to T transition in position 1 of the codon encoding arginine 70 that generates a non-synonymous variant with the amino acid substitution R70C. In order to assess the impact of this SNP on blood pressure, we then analysed this SNP within two

different human cohorts: the UK Biobank (UKBB)[32] and the Genetics Of Diabetes Audit and Research in Tayside (GoDARTS).[33,34]

The UKBB cohort includes ~500,000 individuals with both genetic data and a wealth of phenotypic data, including blood pressure (BP) measurements. Our analysis uses data which was used in our previous genetic analysis of BP in UKBB.[50] For analysis, mean systolic BP (SBP) and diastolic BP (DBP) were calculated from two measurements, adjusted for medication use by adding 15 and 10mmHg to SBP and DBP, respectively, for individuals reported to be taking blood pressure–lowering medication. Following genotyping using a customized array, genetic imputation was performed centrally by UKBB using a reference panel that merged the UK10K and 1000 Genomes Phase 3 panel as well as the Haplotype Reference Consortium (HRC) panel. In addition to central quality control (QC) conducted by UKBB, further QC was performed, including phenotypic QC and restricting to individuals of European ancestry. In order to conduct linear regression analyses for this study, we further restricted to a set of unrelated individuals. Overall, this resulted in a total of 357,151 individuals for analyses of our SNP rs61753924 of interest. The SNP has high quality with imputation quality measure INFO=1.

GoDARTs comprises a cohort of approximately 18,000 individuals half of whom had type 2 diabetes (T2D) at recruitment and half who were free of diabetes. At the time of recruitment, participants gifted a sample of blood for genotyping and consent to link their genetic information to extensive longitudinal electronic medical records. Genotyping in GoDARTS has been previously described.[33] For the present study we made use of the blood pressure records obtained from outpatient clinic visits for the patients with T2D which provides multiple blood pressure records over many years for each individual. Therefore this study was carried out only in the T2D population in GoDARTS. From this longitudinal record we calculated a mean of a maximum of three available blood pressure recordings. Each blood pressure recording was adjusted for the number of antihypertensive medications being prescribed at the time. This was achieved by first determining the average

reduction in blood pressure per antihypertensive drug class obtained from a mixed model analysis using dispensed prescription data available to GoDARTS and then multiplying this value by the number of drug classes being dispensed at the time of each individual blood pressure measurement and adding this product to the blood pressure value.

Statistics:

Quantitative data are shown as means±standard error of the mean (SEM). Differences between experimental groups were tested by one-way or two-way ANOVA followed by a Bonferroni post hoc test or by unpaired t-tests as appropriate. P-values of <0.05 were considered significant. Dose-response curves were fitted using the standard Michaelis-Menton equation (Y=Vmax*X/(Km+X)) and were compared using 2-way ANOVA with changes in V_{max} and K_m considered significant at P<0.05. In *in vitro* physiology studies 2-4 aortic rings were isolated per mouse, multiple observations were combined and the n number is defined as the number of mice not the number of observations.

Western blots that were quantitatively compared with multiple control and test samples all run on the same resolving gel. The average densitometric value for the multiple control samples (from the same resolving gel) was defined and all individual bands (including the control samples) were normalized to this average hence providing an SEM for control and test samples. PLM phosphospecific antibody bands were normalized to total PLM bands within the same gel.

Linear regression analyses of the UKBB data were performed using the R statistical software. The SNP rs61753924 genotype was coded according to a dominant model with carriers of the minor allele T having genotypes CT or TT being compared to individuals with genotype CC as the reference. Analyses were adjusted for age at recruitment and gender. Subgroup analyses were also performed with stratification by gender and/or age groups. P<0.05 was considered to be statistically significant.

Analysis in the GoDARTS was undertaken using STATA in a similar manner to the analysis performed in UKBB with adjustment for mean age at BP recording and also age at recruitment into GoDARTS. Comparisons not indicated were either not significant or not tested.

ADDITIONAL FIGURES

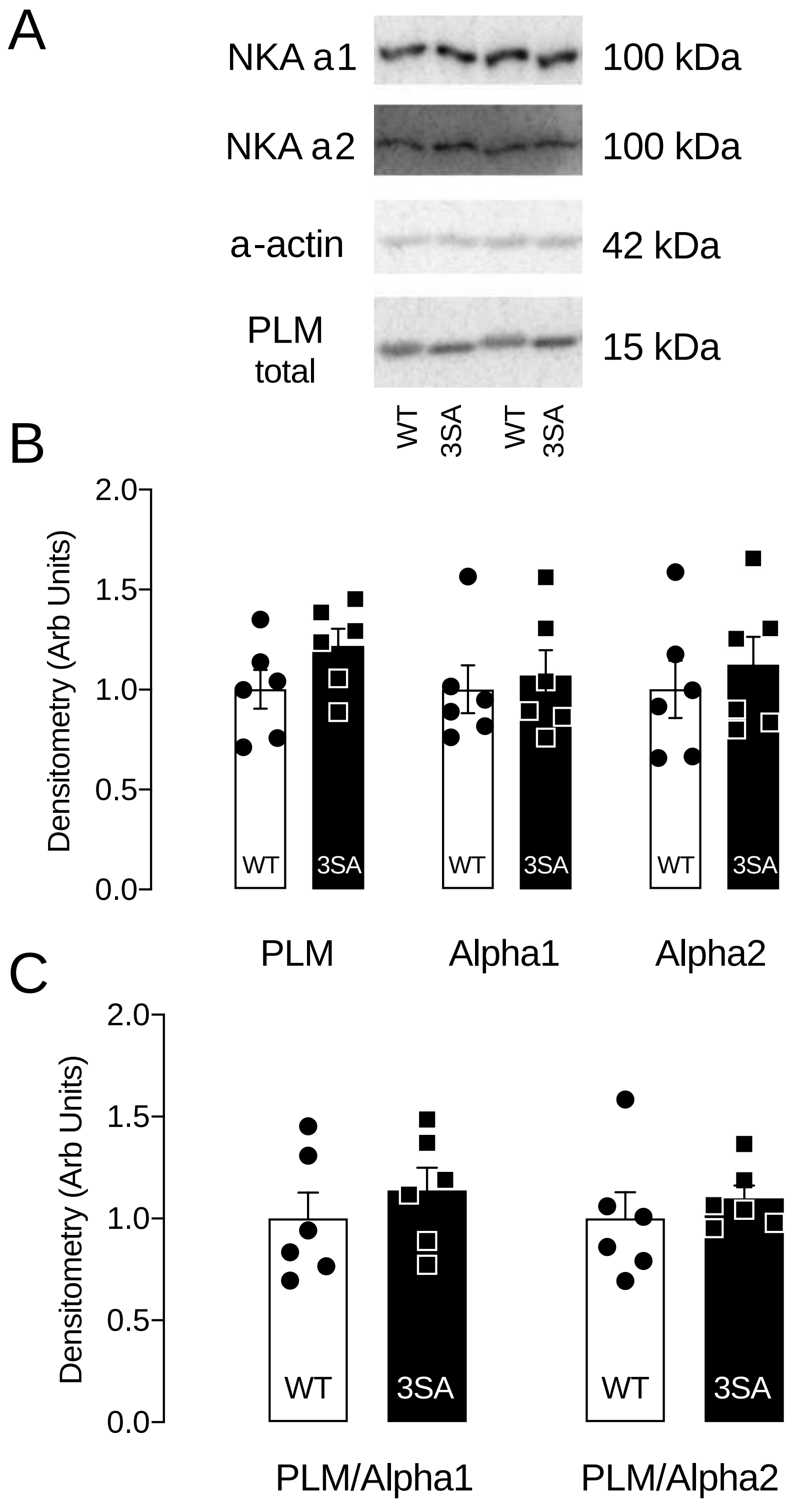

Supplemental Figure I: **Na/K ATPase (NKA) subunit and phospholemman (PLM) expression in aortic homogenates is unaffected by genotype in WT and PLM^{3SA} (3SA) mice. A:** Representative blots for NKA pump complex subunits with α-actin as a loading control. **B:** Mean expression of PLM and α1 and α2 subunits of the NKA complex by genotype. Data are normalized to the mean blot density of the WT expression in each case. **C:** Ratio of PLM to NKA α1 and PLM to NKA α2 by genotype. n=6 (WT) and n=6 (PLM^{3SA}). In Panels B and C unpaired t-test was used for comparison. None of the comparisons were significantly different.

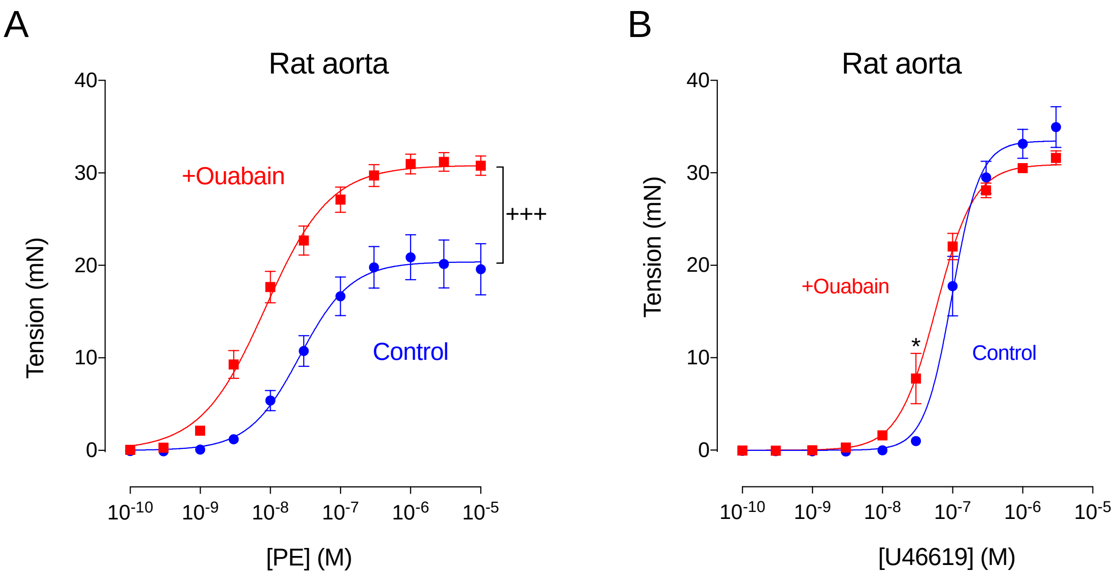

Supplemental Figure II: **Pretreatment of rat aortic smooth muscle with ouabain mimics agonist responses in the PLM^{3SA} mouse aorta. A:** Phenylephrine-induced constriction in rat aortae was potentiated by pre-incubating vessels in ouabain (0.3mmole/L). (n=8/group, +++P<0.001 2-way ANOVA for V_{max} comparison). **B:** Constriction of rat aortae to U46619 was largely unaffected by pretreatment with ouabain (0.3mmoles/L). However, a small but significant ouabain-sensitive limitation of constriction was seen in control rat aortae at U46619 concentrations below 30nmoles/L. *P<0.01, 2-way ANOVA followed by t-test with Bonferroni correction for multiple comparisons.

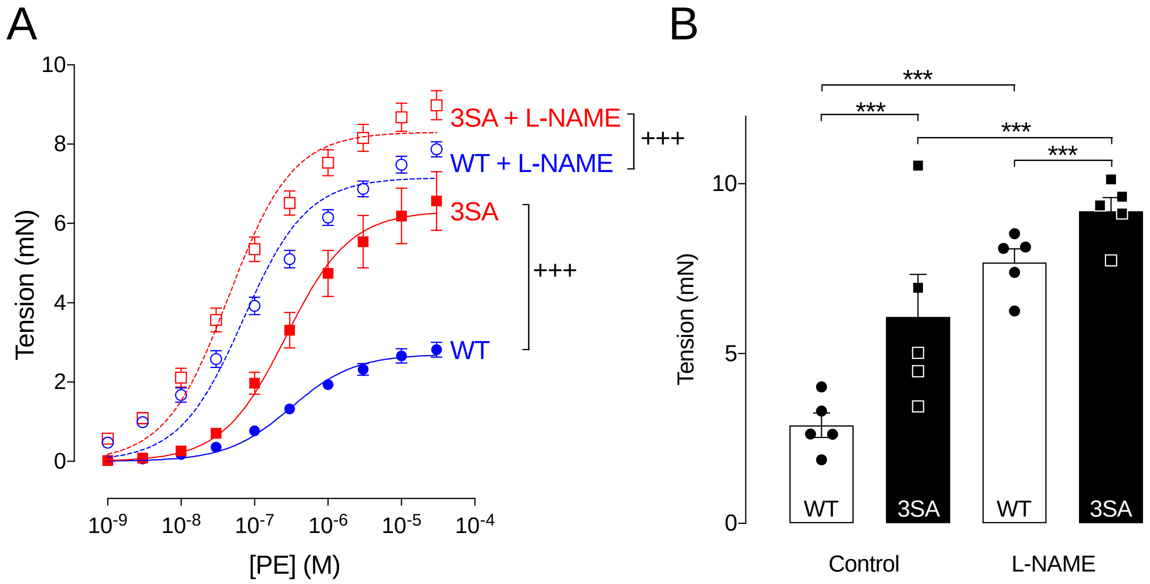

Supplemental Figure III: **The NOS inhibitor L-NAME increased phenylephrine-induced vasoconstriction in both PLM^{3SA} (3SA) and wild-type aortic rings (A).** The limitation of PE-induced constriction (see also Figure 1A) was repeated in a separate series of experiments and, when these aortic rings were subsequently treated with L-NAME (300µmol/L), this ouabain-sensitive difference in constriction between WT and 3SA aortae was partially but not completely blocked (**A** and **B**). Panels **A and B**: n=5/group +++P<0.05 for V_{max} comparison (2-way ANOVA). Panel **B** shows the Influence of genotype and L-NAME pretreatment on maximal-PE-induced vasoconstriction (n=5/group, ***P<0.001 (1-way ANOVA with Bonferroni post-hoc test).

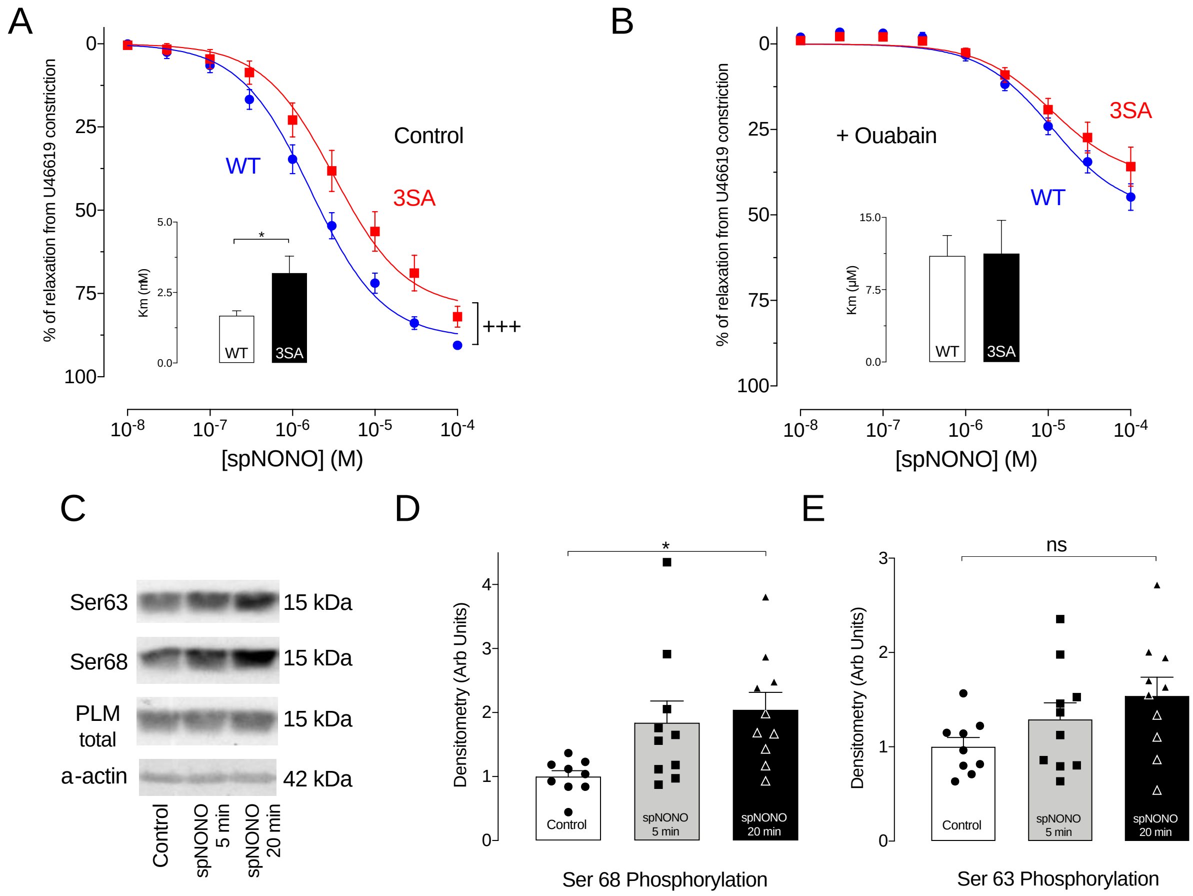

Supplemental Figure IV: **Vasodilation in response to the NO donor spermine nonoate (spNONO) was enhanced in aortae from wild-type compared to PLM^{3SA} mice. A**: Phosphorylation of PLM (as shown in panels **C, D** and **E**) shifted the spNONO dose response to the left and decrease the K_m from 3.19±0.59µM to 1.68±0.17µM (n=5/group, +++P<0.05 for K_m comparison, 2-way ANOVA and *P<0.05 1-way ANOVA with Bonferroni post hoc test). **B:** This shift in the spNONO dose-response curve was completely blocked by pretreatment of aortae with ouabain (100µmoles/L) (n=5/group). Ouabain also reduced the maximal relaxation induced by spNONO in both genotypes. **C:** shows a representative Western blot and mean changes in Ser 68 and Ser 63 are shown in Panels **D** and **E** (n= 10, *P<0.05, all other comparisons to Control not indicated are not significant).

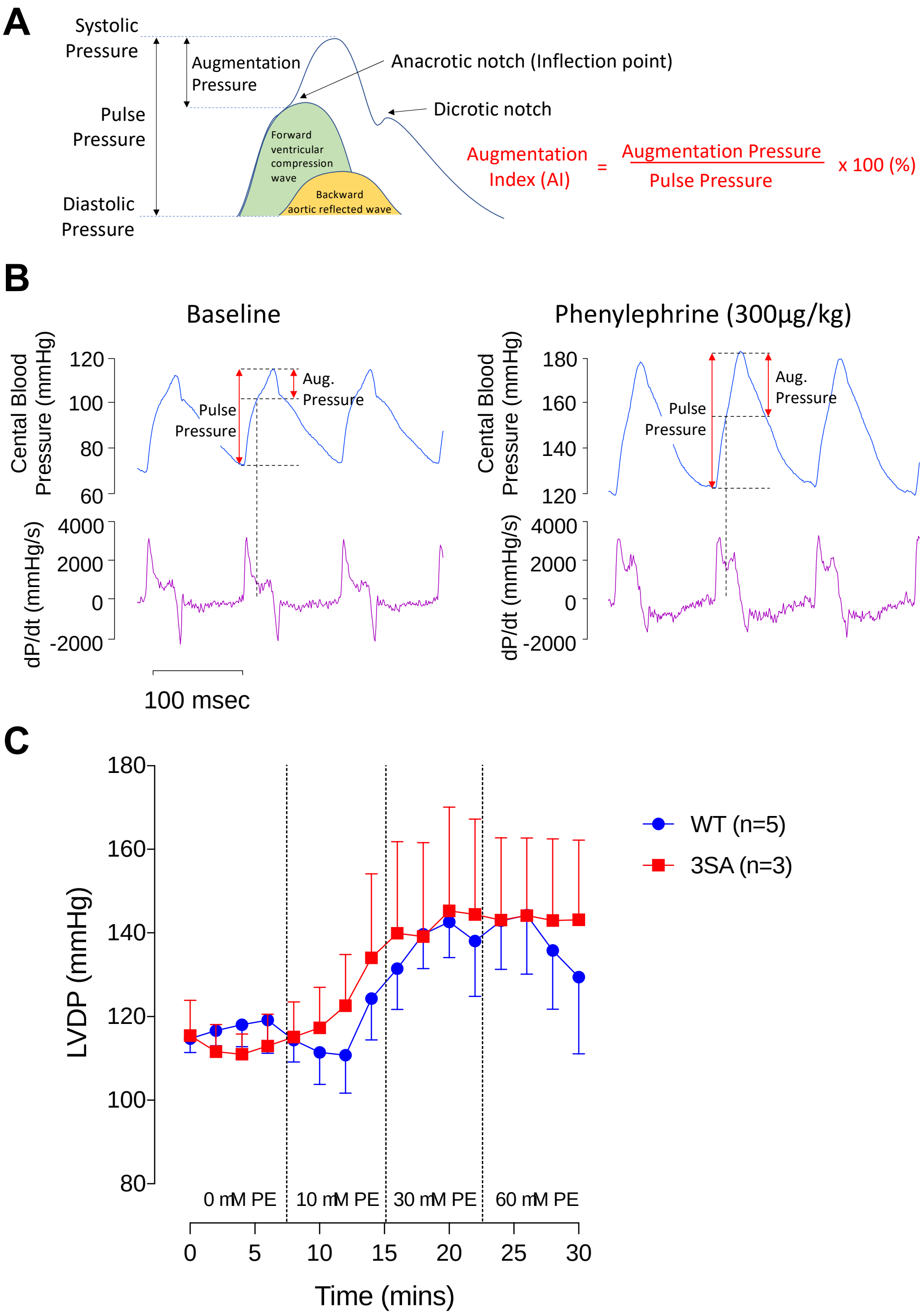

Supplemental Figure V: **Measurement of Augmentation Index (AI) to assess aortic compliance**. **A:** Diagram showing measurement of AI which was measured by identifying the anacrotic notch in the ascending arterial pressure wave and determining the absolute difference between this and peak systolic pressure. The height of the anacrotic notch is primarily determined by the forward compression wave from ventricular contraction while the height of the systolic pressure peak is the sum of this forward compression wave and the backward reflected wave (principally, but not exclusively, from aortic reflection). **B:** Chart Recordings showing aortic pressure (top trace) and dP/dt (lower trace) and how the dP/dt can be used to identify the anacrotic notch during baseline recording and during bolus infusion of phenylephrine (PE). PE enhances AI. **C:** Left-ventricular Developed Pressure (LVDP), measured via and intraventricular balloon in isolated hearts Langendorff perfused at constant perfusion pressure, showing no differences in cardiac contractile responses to PE between genotypes. **Note:** the relationship between cardiac contractility and AI has been shown to be remarkably flat and dominated by aortic compliance.[53] Thus, the enhanced PE-induced elevation of AI in PLM^{3SA} mice (see Figure 2A) is therefore assumed to reflect enhanced aortic stiffness.

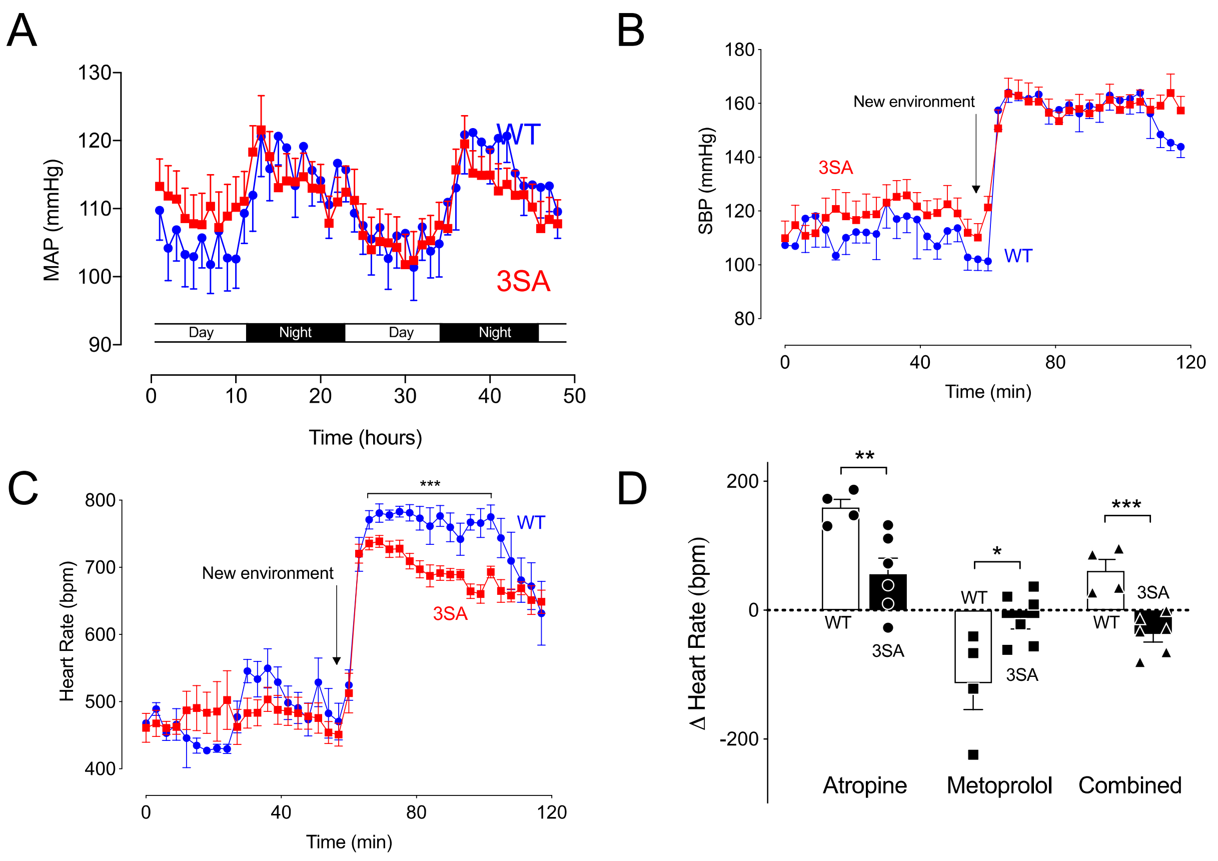

Supplemental Figure VI: **Blood pressure and heart rate changes in conscious PLM^{3SA} (3SA) and wild-type (WT) telemetered mice over 48 hours (A) and during non-restraint stress (B,C), and in anaesthetised mice following bolus infusion of blockers of autonomic nervous system (D). A:** 48 hours recording of mean arterial blood pressure (MAP) in adult (14-16 weeks) WT and PLM^{3SA} mice. Systolic blood pressure (SBP) (**B**) and heart rate changes (**C**) in WT and PLM^{3SA} mice during a non-restraint stress (being moved to a new cage – 'New Environment'). Comparison between WT and 3SA using 2-way ANOVA, overall genotype effect P<0.001. **D:** Heart rate changes in response to bolus IP atropine (2mg/kg) or metoprolol (8mg/kg) or a combination of both. Heart rate changes were measured at steady-state 20 mins after drug infusion. (n= 6-8) and *P<0.05, **P<0.01, ***P<0.001). In panel A n=8 (WT) and n=6 (PLM^{3SA}); panels B-D n=6 (WT, PLM^{3SA}).

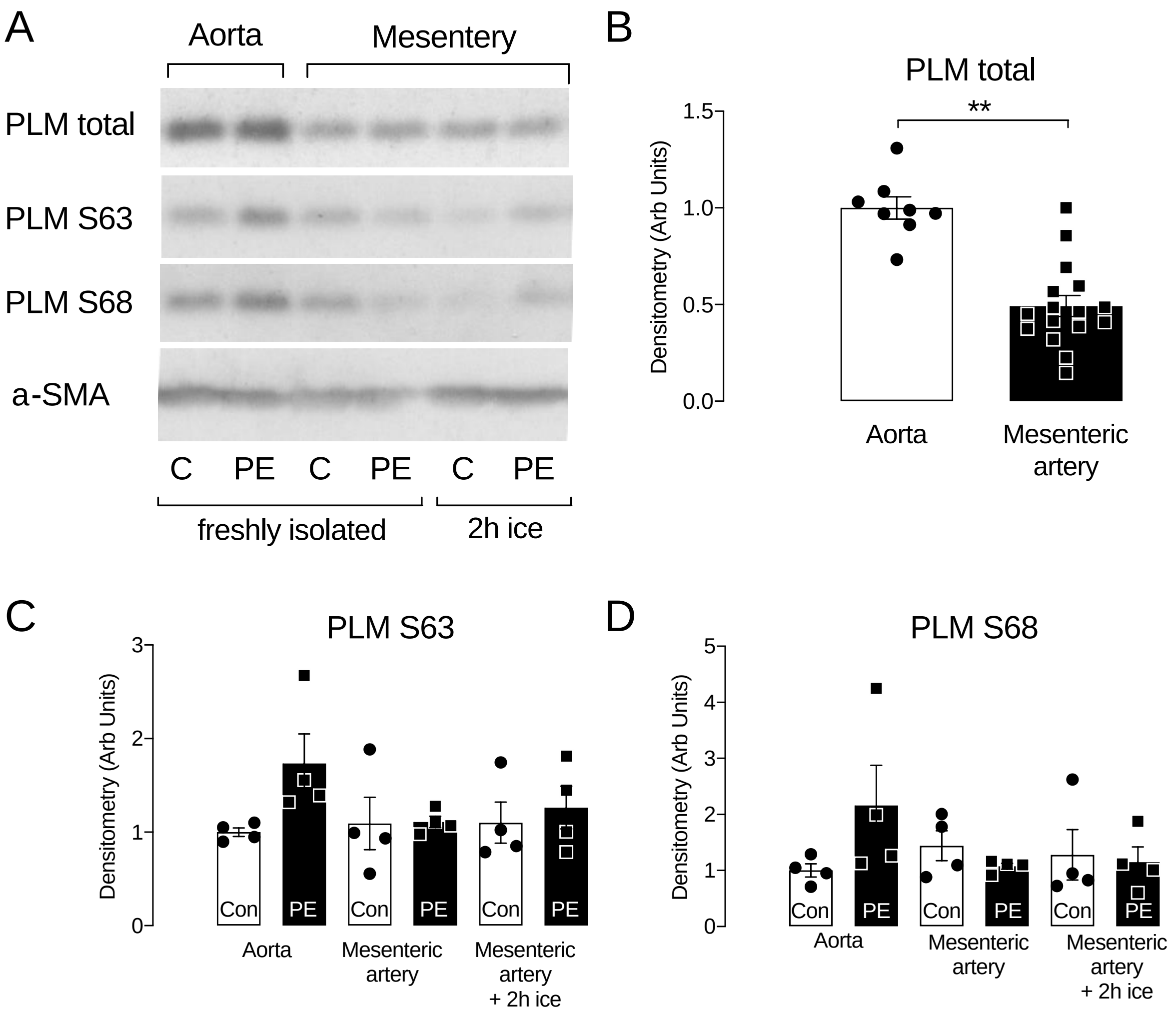

***Supplemental Figure VII:* Phenylephrine (10 µmol/L) phosphorylated phospholemman (PLM) in aortic but not mesenteric smooth muscle *in vitro*.** Data shown are for freshly isolated vessels and mesenteric vessels isolated and stored on ice for 2 hours prior to analysis. **A:** Representative blots and **B:** Total PLM normalized to α-smooth muscle actin. **C:** and **D:** PLM S63 and S68 phosphorylation. In mesentery basal S68 phosphorylation was not detectable above background. Storage of mesenteric vessels on ice prior to analysis did not affect PLM phosphorylation or lack of response to PE (n= 4/grp). In Panels B, C and D unpaired t-test was used for comparison. **P<0.01. Data from aorta are included as positive controls however in this analysis, the PE-induced phosphorylation did not reach the level of statistical significance due to the small sample size (but see data in Figure 1 main manuscript).

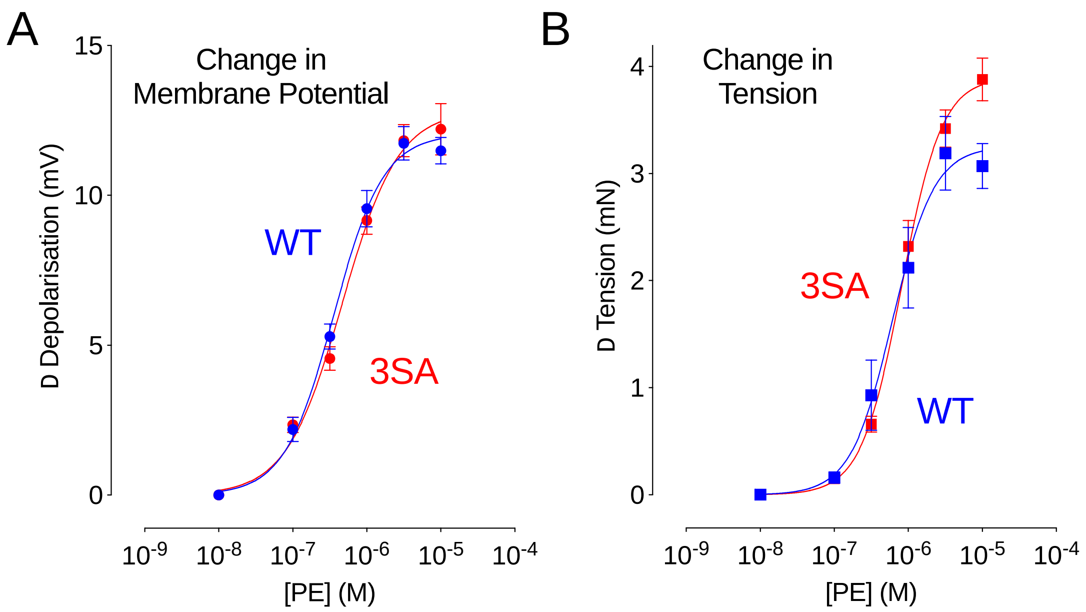

Supplemental Figure VIII: **Despite differences in resting membrane potential (see main manuscript) the *relative* change in membrane potential (A) and *relative* change in tension (B) in response to phenylephrine is similar in WT and 3SA mesenteric vessels.** This is consistent with PE in mesentery inducing no additional phosphorylation of PLM (see Supplement Figure VII). **Note:** any differences in resting tone between genotypes is masked by the normalization of the tension recording at baseline.

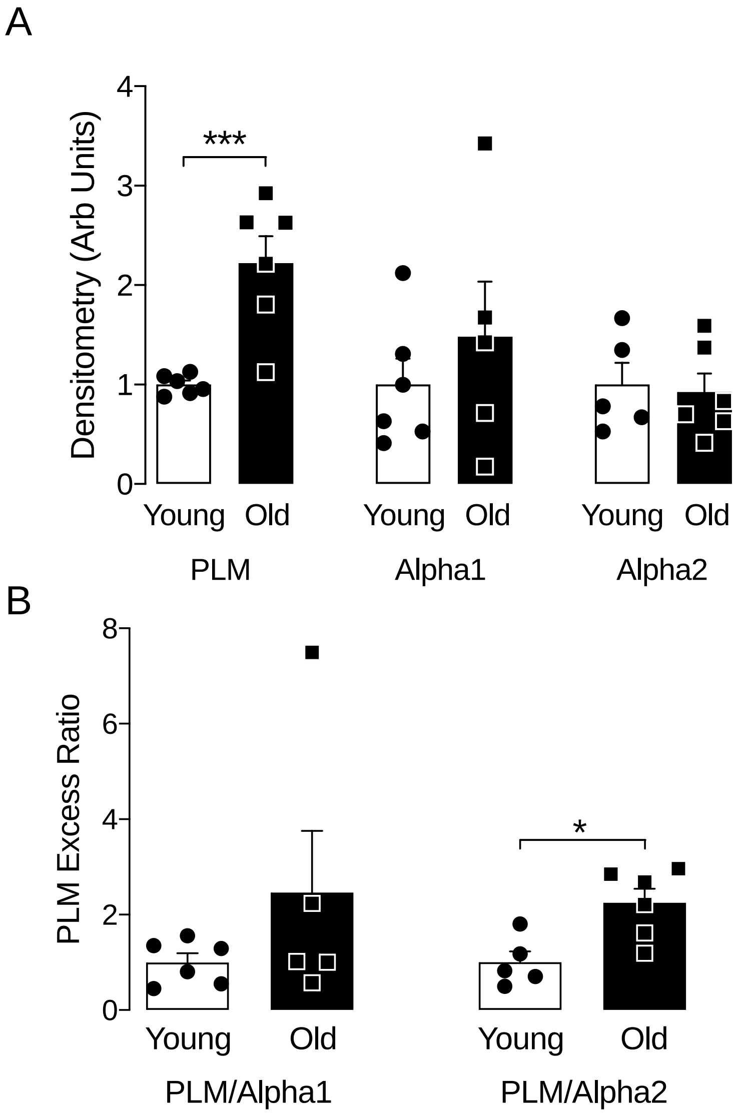

Supplemental Figure IX:* Effect of ageing on phospholemman (PLM) and Na/K ATPase subunit expression in aortae isolated from young and old wild-type mice**. **A:** Total PLM and Na/K ATPase $\alpha 1$ and $\alpha 2$ subunit expression normalized to mean expression measured in young adult samples. Smooth muscle α actin was used as a loading control throughout. **B:** Excess PLM measured as the ratio of total PLM to $\alpha 1$ and $\alpha 2$ subunits. ***Note: Total PLM expression was substantially increased in old mice while there were no significant differences in $\alpha 1$ and $\alpha 2$ expression induced by age. This resulted in a relative excess of PLM over $\alpha 2$ in old animals. In Panels A and B unpaired t-test was used for comparison. n=5-6/group.*P<0.05, ***P<0.001.

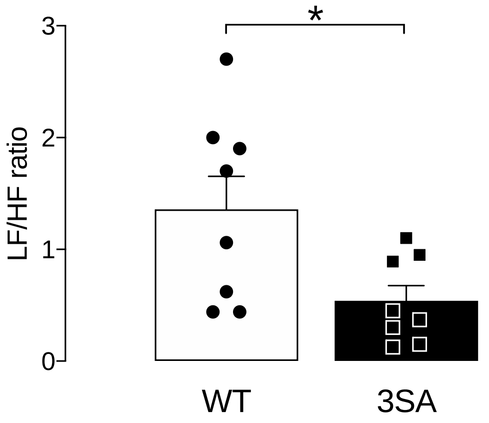

***Supplemental Figure X:* Heart rate variability measured in old wild-type (WT) and PLM^{3SA} (3SA) mice.** The Low/High frequency component (Sympathetic dominance) of Heart Rate Variability (HRV) was measured in conscious telemetered mice under control conditions. n=5/group. Un-paired t-test *P<0.05.

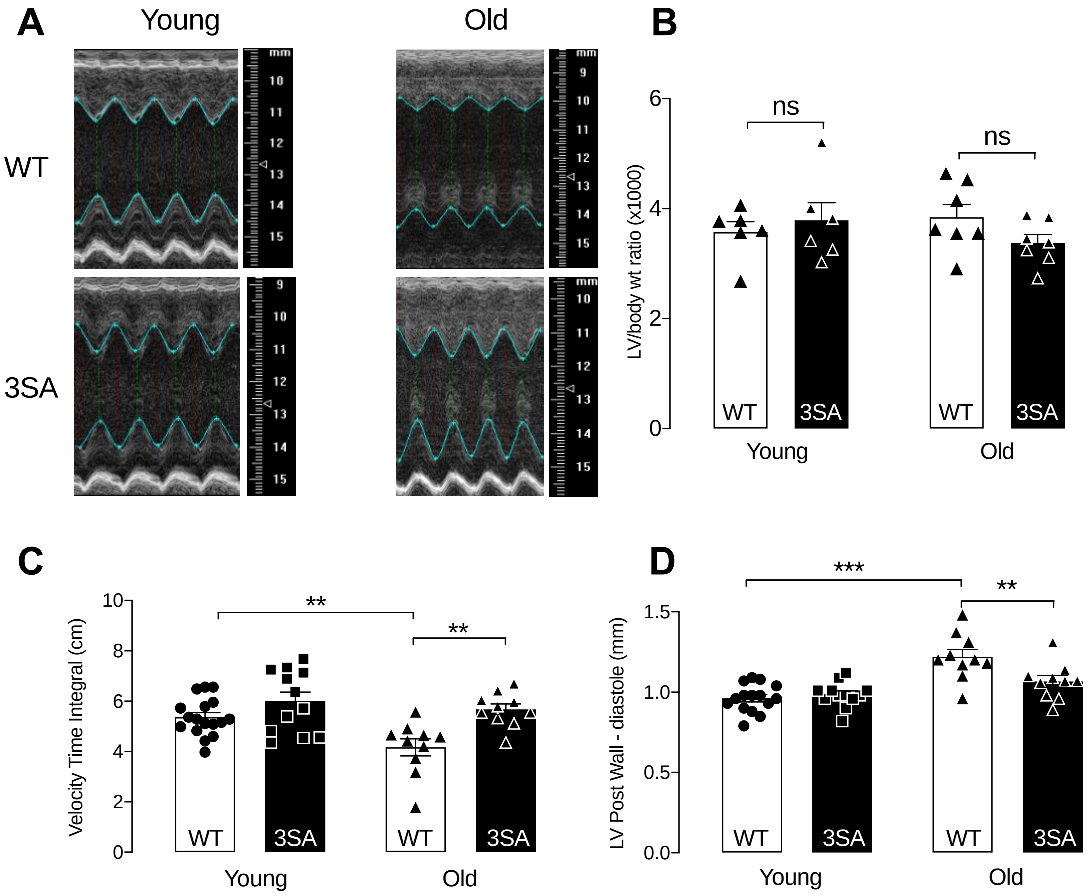

***Supplemental Figure XI:* Effect of ageing on cardiac function and morphometry in young and old WT and PLM^{3SA} (3SA) mice**. **A:** Representative echocardiographic images (M-mode) in young (14-16 weeks) and old (57-60 weeks) WT and PLM^{3SA} (3SA) mice. Age and genotype-dependent changes in LV/body weight ratio: n=6 (young, WT and PLM^{3SA}), n=7 (old, WT and PLM^{3SA}). (**B**), velocity time integral (C) and left-ventricular posterior wall thickness (**D**). Echo measurements were made in anaesthetised mice: In Panels B, C and D comparisons were made using 1-way ANOVA with Bonferroni correction. n=17 (young, WT), n=10 (old, WT), n=12 (young, PLM^{3SA}), n=10 (old, PLM^{3SA}), **P<0.01, ***P<0.001, ns = not significant.

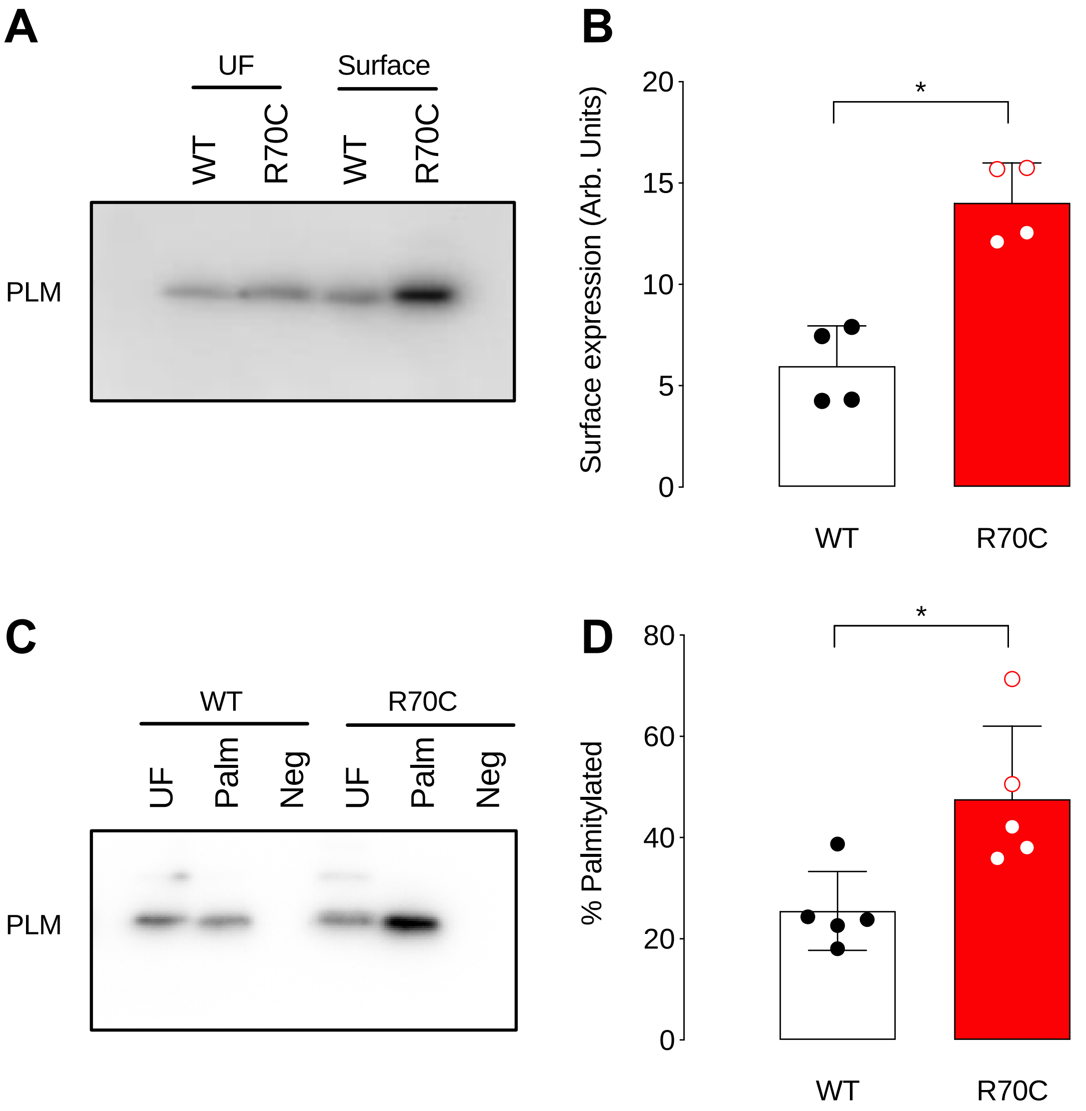

***Supplemental Figure XII*: The R70C mutation of PLM in HEK cells shows increased surface expression and increased fractional palmitoylation. A and B:** Cell surface localisation of WT and R70C PLM (UF = unfractionated). **C and D:** Palmitoylation of WT and R70C PLM. (Palm: palmitoylated proteins). Palmitoylation was measured as previously described.[54] *P<0.05 unpaired t-test.

ADDITIONAL TABLES

Supplemental Table I: Descriptive summary statistics of included participants from the UK Biobank and GoDARTS cohorts.

UKBB	***All***	***Males***	***Males (45 to 55)***
Sample Size, N	357,151	159,204	44,995
SNP genotype (CC/CT/ TT)	350,037 / 7,074 / 40	156,030 / 3,160 / 14	44,107 / 881 / 7
SNP minor allele (T) carrier (N, %)	7,114 (1.99%)	3,174 (1.99%)	888 (1.97%)
Sex (N males, %)	159,204 (44,6%)	159,204 (100%)	44,995 (100%)
Age in yrs ([min-max], mean ± SD)	[40-70], 56.4 ± 8.0	[40-70], 56.5 ± 8.1	[45-55], 49.7 ± 2.9
SBP# (mean ± SD) (mmHg)	140.5 ± 20.5	144.2 ± 19.2	138.5 ± 16.7
DBP# (mean ± SD) (mmHg)	84.1 ± 11.2	86.4 ± 11.0	85.7 ± 10.9
Hypertensives (N, %)*	179,242 (50.2%)	92,021 (57.8%)	21,101 (46.9%)
taking anti-HTN meds (N, %)	61,607 (17,2%)	31,180 (19.6%)	4,800 (10.7%)
BMI (mean ± SD)	27.2 ± 4.7	27.7 ± 4.2	27.7 ± 4.3
Diabetes (N, %)	14,362 (4.0%)	8,458 (5.3%)	1,561 (3,5%)

GoDARTS	***All***	***Males***	***Males (40-65)***
Sample size, N	7,784	4,460	1,786
SNP genotype (CC/CT/TT)	7,545/2,37/2	4,276/130	1,742/44
SNP minor allele (T) carrier(N, %)	239 (3.07)	130(3.0%)	44 (2.46%)
Sex (N males, %)	4,406(56%)	100%	100%
Age in yrs ([min-max], mean ± SD)	[23-94], 66.7±10.7	[23-94], 66.4±10.4	[40-65], 57.0±5.9
SBP# (mean ± SD) (mmHg)	143.0 ±11.3	142.0±11.0	140.6±11.0
DBP# (mean ± SD) (mmHg)	79.7±6.3	80.0±6.5	82.6±6.2
*Hypertensive * (N, %)*	4,819 (61.9%)	2,594(58.9)	950(53.2%)
Taking anti-HTN meds (N, %)	7,000(89.9%)	3,976(90.2%)	1,554(87%)

#BP traits adjusted for anti-HTN medication use (+15mmHg for SBP; +10mmHg for DBP)
*Hypertension defined as adj-SBP≥140mmHg or adj-DBP≥90mmHg
